# Use of physiological based pharmacokinetic modeling for cross-species prediction of pharmacokinetic and tissue distribution profiles of a novel niclosamide prodrug

**DOI:** 10.3389/fphar.2023.1099425

**Published:** 2023-04-11

**Authors:** Mengbi Yang, Amy Q. Wang, Elias C. Padilha, Pranav Shah, Natalie R. Hagen, China Ryu, Khalida Shamim, Wenwei Huang, Xin Xu

**Affiliations:** Division of Preclinical Innovation, National Center for Advancing Translational Sciences, National Institutes of Health, Rockville, MD, United States

**Keywords:** niclosamide, prodrug, PBPK modeling, cross-species prediction, tissue distribution

## Abstract

**Introduction:** Niclosamide (Nc) is an FDA-approved anthelmintic drug that was recently identified in a drug repurposing screening to possess antiviral activity against SARS-CoV-2. However, due to the low solubility and permeability of Nc, its *in vivo* efficacy was limited by its poor oral absorption.

**Method:** The current study evaluated a novel prodrug of Nc (PDN; NCATS-SM4705) in improving *in vivo* exposure of Nc and predicted pharmacokinetic profiles of PDN and Nc across different species. ADME properties of the prodrug were determined in humans, hamsters, and mice, while the pharmacokinetics (PK) of PDN were obtained in mice and hamsters. Concentrations of PDN and Nc in plasma and tissue homogenates were measured by UPLC-MS/MS. A physiologically based pharmacokinetic (PBPK) model was developed based on physicochemical properties, pharmacokinetic and tissue distribution data in mice, validated by the PK profiles in hamsters and applied to predict pharmacokinetic profiles in humans.

**Results:** Following intravenous and oral administration of PDN in mice, the total plasma clearance (CL_p_) and volume of distribution at steady-state (Vd_ss_) were 0.061–0.063 L/h and 0.28–0.31 L, respectively. PDN was converted to Nc in both liver and blood, improving the systemic exposure of Nc in mice and hamsters after oral administration. The PBPK model developed for PDN and *in vivo* formed Nc could adequately simulate plasma and tissue concentration-time profiles in mice and plasma profiles in hamsters. The predicted human CL_p_/F and Vd_ss_/F after an oral dose were 2.1 L/h/kg and 15 L/kg for the prodrug respectively. The predicted Nc concentrations in human plasma and lung suggest that a TID dose of 300 mg PDN would provide Nc lung concentrations at 8- to 60-fold higher than *in vitro* IC_50_ against SARS-CoV-2 reported in cell assays.

**Conclusion:** In conclusion, the novel prodrug PDN can be efficiently converted to Nc *in vivo* and improves the systemic exposure of Nc in mice after oral administration. The developed PBPK model adequately depicts the mouse and hamster pharmacokinetic and tissue distribution profiles and highlights its potential application in the prediction of human pharmacokinetic profiles.

## 1 Introduction

Niclosamide (Nc) is an FDA-approved anti-tapeworm drug for use in humans to treat tapeworm infections. It is also included on the World Health Organization’s list of essential medicines. Nc exerts its anti-tapeworm effect by inhibiting oxidative phosphorylation and stimulating adenosine triphosphatase activity in the mitochondria (Jeon et al., 2020; Xu et al., 2020; Shamim et al., 2021). A recent drug repurposing screening has suggested that Nc possesses potent antiviral activity against viruses including Zika virus, MERS-CoV, SARS-CoV, SARS-CoV-2, hepatitis C virus, and human adenovirus (Jeon et al., 2020; Xu et al., 2020; Shamim et al., 2021). It was recently demonstrated that Nc inhibits SKP2 activity, which enhances autophagy and reduces MERS-CoV replication, and a similar mechanism is expected to contribute to the inhibition of SARS-CoV-2 infection (Jeon et al., 2020). Nc exhibits very potent antiviral activity against SARS-CoV-2 with IC_50_ ranges from 0.06–0.28 μM (Weiss et al., 2021).

Due to its low aqueous solubility, low permeability, and extensive liver and intestine metabolism, Nc has very low oral bioavailability. The solubility of Nc is reported to range from 0.2 to 19 μg/mL, depending on the temperature and pH conditions as well as on salt contents (Andrews et al., 1982). The solubility of the ethanolamine salt of Nc can be improved up to 372 μg/mL (Andrews et al., 1982). Nc also displays poor permeability with the parallel artificial membrane permeability assay (PAMPA) permeability of less than 1 × 10^−6^ cm/s (Shamim et al., 2021). Pharmacokinetic (PK) study in rats that received a single oral (PO) dose of 50 mg/kg ^14^C-radiolabeled Nc indicated that about one-third of the dose was absorbed while two-thirds of the dose was excreted in feces (Andrews et al., 1982). The oral bioavailability of Nc in rats and dogs is only 5.5% and 0.54% respectively (Choi et al., 2021).

Nc has been investigated in multiple clinical trials for colorectal cancer, prostrate carcinoma, and COVID-19. In an early human clinical study, male and female volunteers received a single oral dose of 2000 mg ^14^C-labeled Nc. The fraction of ^14^C-activity eliminated with the urine was 2–25% over a 4-day period. The remainder was eliminated in feces. Serum maximal concentration (C_max_) equivalent to 0.25–6.0 μg/mL Nc was detected (Andrews et al., 1982). A recent Phase I dose-escalation study reported that oral dose of Nc could not be escalated above 500 mg three times a day (TID). The maximum plasma concentrations could not achieve the threshold to inhibit cell growth in castration-resistant prostate cancer models (Schweizer et al., 2018). A prospective phase II clinical trial of Nc in patients with metastasized colorectal cancer indicated that an oral dose of 2 g once a day (QD) per patient could produce a median plasma C_max_ of 0.665 μg/mL (Burock et al., 2018), which is still modest for anticancer treatment. In a recently published phase Ib clinical trial of reformulated Nc, an oral dose of 1.2 g TID Nc could achieve a mean peak plasma concentration of 0.159 μg/mL in three male prostate cancer patients (Parikh et al., 2021). Multiple clinical trials for the treatment of COVID-19 and cancer with Nc are still ongoing.

**FIGURE 1:**
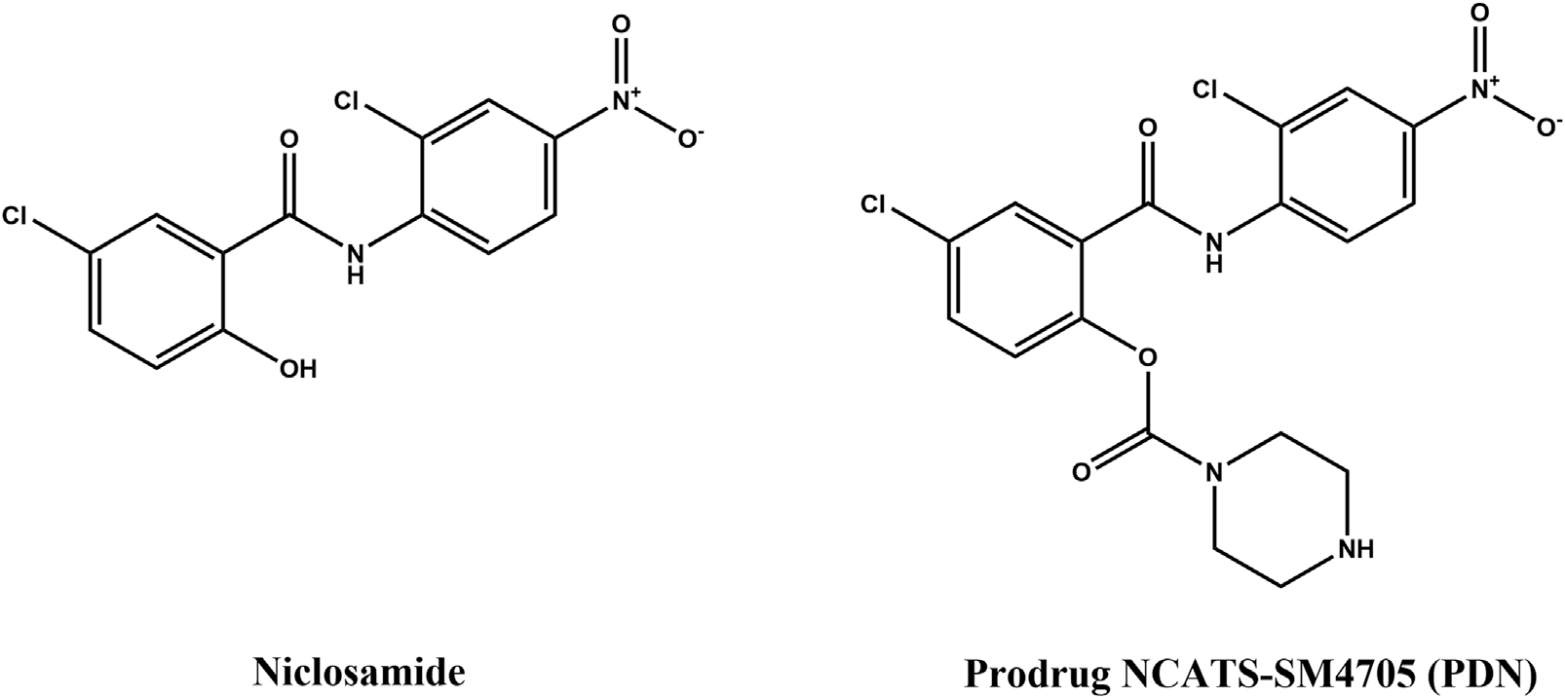
Structures of niclosamide (Nc) and its prodrug NCATS-SM4705 (PDN).

The low solubility, poor bioavailability, and poor plasma exposure of Nc hinder its use in patient treatments (Cairns et al., 2022). Previous efforts have been made to increase its systemic exposure by optimizing injectable formulation for IV administration (Ye et al., 2015) or to increase its local concentrations at the lung by developing inhalation or intranasal formulations (Backer et al., 2021; Jara et al., 2021). However, oral therapeutics have the potential to maximize patient benefit due to their non-invasiveness, patient compliance, and convenience. It is still the first choice of route of administration, especially during a pandemic. In order to achieve high exposures after oral delivery, ester-derivative prodrugs were employed. Few attempts have been made in this approach. An Nc stearate prodrug has been developed for increasing its solubility but was only evaluated for IV administration (Reddy et al., 2020). In another study, five prodrugs of Nc were synthesized, and the *in vitro* and *in vivo* efficacy was evaluated (Elkihel et al., 1994). However, the pharmacokinetic profiles were not determined for these prodrugs. In this study, we evaluated *in vitro* ADME properties and pharmacokinetics (PK) of an Nc prodrug, namely, NCATS-SM4705, in lab animals. The structures of Nc and its prodrug NCATS-SM4705 (PDN) are shown in Figure 1. PDN showed *in vitro* activity against SARS-CoV-2 in our previous cytopathic effect assay and cytotoxicity counter screen; and *in vivo* efficacy was tested in a hamster model of SARS-CoV2 infection (Huang et al., 2022).

To predict human pharmacokinetic profiles, modeling methodologies integrating both *in vitro* absorption, distribution, metabolism, and excretion (ADME) properties and preclinical pharmacokinetic data were applied. Physiologically based pharmacokinetic (PBPK) modeling combines drug information and animal physiology at organ levels and provides mechanistic representations of drug concentrations in biological systems (Bi et al., 2016; Kuepfer et al., 2016). This system pharmacology approach can not only predict the pharmacokinetic profiles of a new chemical entity in various tissues but also analyze the *in vivo* ADME processes with advanced scientific knowledge at the mechanistic level, supporting further drug candidate optimization and development, and guiding dose selection in clinical trials.

The current study aims to evaluate a novel prodrug of Nc, NCATS-SM4705 (PDN), on improving the *in vivo* exposure of Nc. *In vitro* ADME properties and PK of PDN were studied in mice and hamsters. Based on *in silico*, *in vitro* and *in vivo* mouse data, a whole-body PBPK model was developed to characterize the concentration-time profiles of PDN and Nc in systemic circulation and different tissues. The PBPK model was verified using hamster PK profiles and then extrapolated to predict the concentration-time profiles in humans. The simulation was performed at different dose regimens for first-in-human PK prediction and future drug development guidance.

## 2 Materials and methods

### 2.1 Materials

Nc was purchased from Sigma-Aldrich, Inc. NCATS-SM4705 (PDN) synthesized by NCATS chemists (Huang et al., 2022) with purity>97% was used. Liver microsomes and cytosolic fractions from mice, hamsters, and humans were purchased from Sekisui XenoTech (Kansas City, KS, United States). NADPH regenerating system and UGT reaction mix were obtained from Corning Gentest (Corning, NY, United States). Human and mouse blank plasma was purchased from BioIVT (Westbury, NY, United States). Hamster blank control plasma was collected before dosing in the hamster pharmacokinetic studies.

### 2.2 *In vitro* ADME assays

#### 2.2.1 Kinetic aqueous solubility

Pion’s patented µSOL assay was used for kinetic solubility determination as described previously (Sun et al., 2019). Test compounds (10 mM) were prepared in dimethyl sulfoxide (DMSO) and diluted to a final drug concentration of 150 µM in the aqueous solution (pH 7.4, 100 mM Phosphate buffer). Samples were incubated at room temperature for 6 h and vacuum-filtered using Tecan Te-Vac to remove any precipitates. The concentration of the compound in the filtrate was measured *via* UV absorbance (λ: 250–498 nm). The unknown drug concentration was determined by comparing the fully solubilized reference plate which contained 17 µM of compound dissolved in spectroscopically pure n-propanol. All compounds were tested in duplicates. The kinetic solubility (µg/mL) of compounds was calculated using the µSOL Evolution software. The three controls used were albendazole (low solubility), phenazopyridine (moderate solubility) and furosemide (high solubility).

#### 2.2.2 Parallel artificial membrane permeability assay (PAMPA)

Stirring double-sink PAMPA method (patented by pION Inc.) was employed to determine the permeability of compounds *via* PAMPA as described previously (Sun et al., 2017). pH 7.4 solution was used in both donor and acceptor wells. The test samples (0.05 mM test article in pH 7.4 buffer with 0.5% DMSO) were added in the donor compartment and were stirred using the Gutbox technology (Pion Inc.) to reduce the aqueous boundary layer. The test article concentrations in the donor and acceptor compartments were measured using a UV plate reader (Nano Quant, Infinite 200 PRO, Tecan Inc., Männedorf, Switzerland). The three controls used were ranitidine (low permeability), dexamethasone (moderate permeability) and verapamil (high permeability).

#### 2.2.3 Plasma protein binding

Plasma protein binding was determined with equilibrium dialysis method using the Rapid Equilibration Dialysis (RED) device (Thermo Fisher Catalog # 90006). The assay was performed using manufacturer’s procedures. Briefly, plasma spiked with a test compound (5 µM) was added to the plasma chamber of the RED device. Blank potassium phosphate buffer (100 mM) was added to the buffer chamber of the RED device and the plate was incubated at 37°C. Aliquots of the buffer and the plasma were taken at 0 and 4 h, and the concentrations of free and bound test compounds were determined using the previously described LC-MS method (Shah et al., 2016).

#### 2.2.4 Metabolic stability in plasma and liver

The metabolic stability of PDN and Nc in plasma was determined by incubating 2 µM Nc or 3 µM PDN in 50 μL mouse, hamster, and human plasma at 37°C for up to 120 min, respectively. The metabolic stability and biotransformation of PDN and Nc in liver was determined using liver microsomes. Briefly, the 50 μL incubation system consists of 0.5 mg/mL mouse, hamster, and human microsomal protein, Nc (2 µM) or PDN (0.3–3 µM), NADPH regeneration system (containing 0.650 mM NADP+, 1.65 mM glucose 6-phosphate, 1.65 mM MgCl_2_, and 0.2 unit/mL G6PD), and UGT reaction mix (containing 0.025 mg/mL alamethicin, 8 mM MgCl_2_, and 2 mM UDPGA) in 100 mM phosphate buffer at pH 7.4. The incubation was carried out at 37°C for 15, 30, and 60 min. The reaction in plasma or liver microsomes was quenched by adding 200 μL of cold acetonitrile (containing 100 ng/mL tolbutamide as internal standard). After a 20 min centrifugation at 3000 rpm, 4°C, the supernatant was transferred to an analysis plate and analyzed by UPLC-MS/MS as described in Section 2.4.2.

The apparent *in vitro* clearance (CL_*in vitro*,app_) for Nc depletion in liver microsomes (CL_*in vitro*,app,Nc-total_) and the apparent *in vitro* rate of PDN depletion in plasma (k_*in vitro*,app,PDN-total_) were calculated using the following equation:

$${CL}_{in\;vitro,app}=0.693/\left(t_{1/2}\times mc\right)$$

$$k_{in\;vitro,app,PDN-total}=0.693/t_{1/2}$$

The *mc* was the microsome concentration used in the incubation (0.5 mg/mL). The substrate disappearance half-life (t_1/2_) was estimated by regression analysis of the semi-logarithmic (ln Conc vs*.* time) plots. The *in vitro* clearance of PDN to Nc (CL_*in vitro*,app, PDN-Nc)_ in plasma was calculated from the formation of Nc. The V_max,_ _*in vitro*,app, PDN-total_ and K_m_ for PDN depletion in liver microsomes were estimated by the Michaelis-Menten model using GraphPad Prism version 8.

The values of K_m_, V_max,_ _*in vitro*,app, PDN-total_, and V_max,_ _*in vitro*,app, PDN-Nc_ of PDN in liver microsomes were calculated by fitting the curve of biotransformation from PDN to Nc based on following equations using Berkeley Madonna version 10:

$$\left.dC_{PDN}/dt=-V_{\max,in\;vitro,app,PDN-total}\times mc\times C_{PDN}\;/\;(K_{m}+C_{PDN}\right)$$

$$dC_{Nc}/dt=V_{\max,in\;vitro,app,PDN-Nc}\times mc\times C_{PDN}\;/\;\left(K_{m}+C_{PDN}\right)-{CL}_{in\;vitro,app,Nc}\times mc\times C_{Nc}$$

where $C_{PDN}$ and $C_{Nc}$ are the concentration of PDN and Nc at time t, *mc* was the microsome concentration, and V is the volume of the incubation system. CL _*in vitro*,app, Nc_ is the apparent *in vitro* clearance of Nc determined by its depletion *in vitro* t_1/2_. V_max,_ _*in vitro*,app, PDN-total_ and V_max,_ _*in vitro*,app, PDN-Nc_ are the maximum rates of reaction for the conversion of PDN to total metabolites and to Nc in liver microsomes.

CL_*in vitro*,app_ expressed as mL/min/mg microsomes were scaled to the predicted CL_intrinsic_ using scaling factor (SF), microsome level and liver weight as indicated below:

$${CL}_{intrinsic}={CL}_{in\;vitro,app}\times SF\times \left(liver\;weight\times microsome\;level\;per\;gram\;liver\right)$$

### 2.3 Pharmacokinetics and tissue distribution studies

#### 2.3.1 Mouse PK study

The in-life study (Animal Study Protocol Number: NIH ORS-51) was conducted at the NIH Animal Facility by the Division of Veterinary Resources (DVR, Bethesda, MD). In this study, 25–35 g male C57BL/6 mice (Charles River Laboratories, Frederick, MD) were used. For the IV groups, 66 mice (33 mice per dosing group, 3 mice per timepoint) were used for a single bolus IV dose of 3 or 10 mg/kg of PDN *via* tail vein injection. For the PO group, 30 mice (3 mice per timepoint) received a dose of 10 mg/kg of PDN *via* oral gavage. The IV and PO doses were selected to obtain a complete concentration profile in plasma and tissues, based on a preliminary study. Animals were not fasted in this study. The formulation used was 5% ethanol, 60% PEG-300 and 35% (20% HP-b-CD in water). All dosing solutions were freshly prepared on the day of dosing. At designated time points, blood samples were collected, and mice were euthanized with CO_2_ in accordance with NIH ARAC Guidelines after blood collection. The death was ensured by cervical dislocation or transthoracic puncture. Lung, liver, adipose, brain, heart, small intestine, kidney, skeletal muscle, and spleen samples were obtained from mice. The sampling time points were before dosing and at 0.083, 0.25, 0.5, 1, 2, 4, 7, 24, 30, and 48 h after IV administration and 0.167, 0.5, 1, 1.5, 2, 4, 7, 24, 30, and 48 h after oral administration. Tissue samples were rinsed with cold saline, dried on filtrate paper, weighed, and snap frozen by placing into dry ice after collection. All samples were stored at −80°C until the analysis. All blood samples were collected in K_2_EDTA tubes and centrifuged at 3000 g for 10 min at 4°C to obtain plasma. Plasma samples were transferred to tubes preloaded with NaF (final concentration at 5 mM in 200 µL plasma) and stored at −80°C until analysis.

#### 2.3.2 Hamster study

In this study, 102–126 g male LVG golden Syrian hamsters (Vital River Laboratory Animal Technology Co., Ltd., Beijing, China) were used. For the IV group, a single bolus IV dose of 3 mg/kg of PDN was administered to 24 hamsters (3 hamsters per time point). For the PO group, a dose of 30 mg/kg of PDN was administered *via* oral gavage to 21 hamsters (3 hamsters per time point). The formulation used for both IV and PO was 20% HP-β-CD in water (0.6 mg/mL PDN for IV and 3 mg/mL for PO). All dosing solutions were freshly prepared on the day of dosing. Blood and lung samples were obtained from hamsters at 0.083, 0.25, 0.5, 1, 2, 4, 8, and 24 h after IV administration and 0.25, 0.5, 1, 2, 4, 8, and 24 h after oral administration. After collection, tissues were weighed and frozen. All blood samples were centrifuged at 4°C to obtain plasma. Plasma samples were transferred to tubes preloaded with NaF (final concentration at 5 mM in 200 µL plasma) and stored at −80°C until analysis.

### 2.4 Bioanalytical methods

#### 2.4.1 Sample preparation

Tissue samples were homogenized in water with 5 mM NaF. The tissue to water volume ratio was 1:6 for spleen and lung, and 1:3 for other tissues. For fat tissue, an additional 3-fold volume of acetonitrile was added to the water homogenate and was vortexed in a shaker for another 30 min.

Nc and PDN were extracted from plasma and tissue homogenates using acetonitrile. A 10 μ aliquot of samples, blank controls (blank plasma or tissue homogenates), quality controls (QC), and standards were added into 200 μ acetonitrile solution with 100 ng/mL tolbutamide (internal standard, IS). All samples were then thoroughly shaken for 5 min to precipitate proteins. The samples were centrifuged at 3000 rpm and 4°C for 30 min. The supernatant was injected for UPLC-MS/MS analysis.

#### 2.4.2 UPLC-MS/MS methods

Chromatographic resolution was achieved on a Waters Acquity UPLC BEH C_18_ 1.7 μm (2.1 × 50 mm) column at 60°C. The mobile phase consisted of 0.1% formic acid in water (A) and 0.1% formic acid in acetonitrile (B), the flow rate was 600 μL/min. For the chromatographic gradient, mobile phase A was kept at 95% between and 0.2 min and linearly decreased to 5% at 1.2 min. Mobile phase A was kept at 5% until 1.9 min and set to initial conditions in a 2.1 min total runtime. All analyses were carried out using a Waters Acquity I-Class UPLC interfaced with a Waters Xevo TQ-S mass spectrometer. The mass spectrometer multiple reaction monitoring (MRM) experiment was operated with an ESI source at negative mode with spray voltage of 1 kV, and temperature of 600°C. The m/z of the precursor and product ions monitored for each compound is summarized in Table 1. Both Q1 and Q3 were set at unit resolution. No endogenous components interfered with the analysis of PDN and Nc in samples. Concentrations were determined by a weighted (1/concentration^2^) least-squares linear regression method. The correlation coefficients of the calibration curves were all greater than 0.99. The precision of QCs (*n* = 4–6) were within 15% except for LLOQs which were within 20%. The accuracy (%) of QCs (*n* = 4–6) was within 85%–115% except for LLOQs which were within 80%–120%. The linear range, precision and accuracy of QCs in various tissues and plasma are shown in Supplementary Table S1 and Supplementary Table S2.

**TABLE 1:** MRM transitions monitored for the analysis of Nc and PDN in biological samples.

Compound	Parent m/z	Product m/z	Cone voltage (V)	Collision energy (eV)	Dwell (s)
Nc	325	171	25	26	0.017
Nc	325	289	25	17	0.017
Nc	325	135	25	38	0.017
PDN	437	283	25	16	0.017
Tolbutamide (IS)	269	170	25	17	0.017

### 2.5 Pharmacokinetic analysis

#### 2.5.1 Non-compartmental analysis

Phoenix WinNonlin, version 8.2 (Certara, MO) was used to perform pharmacokinetic analysis with the non-compartmental approach (Models 200 and 201 for PO and IV datasets). The area under the concentration vs. time curve (AUC) was calculated using the linear trapezoidal method. The terminal rate constant (*λ*) was obtained from the slope of at least three data points of the apparent terminal phase of the log linear concentration vs. time curve. AUC_0–∞_ was estimated as the sum of the AUC_0–t_ (where t is the time of the last measurable concentration) and Ct/λ. The apparent terminal half-life (t_1/2_) was calculated as 0.693/λ. Bioavailability (F) was calculated using ratios of dose-normalized AUC of PO and IV administration. After administration of PDN, the dose-normalized AUC of Nc is calculated using the equivalent Nc dose. For example, for a 10 mg dose of PDN, the equivalent dose of Nc is 7.43 mg.

#### 2.5.2 PBPK modeling

**FIGURE 2:**
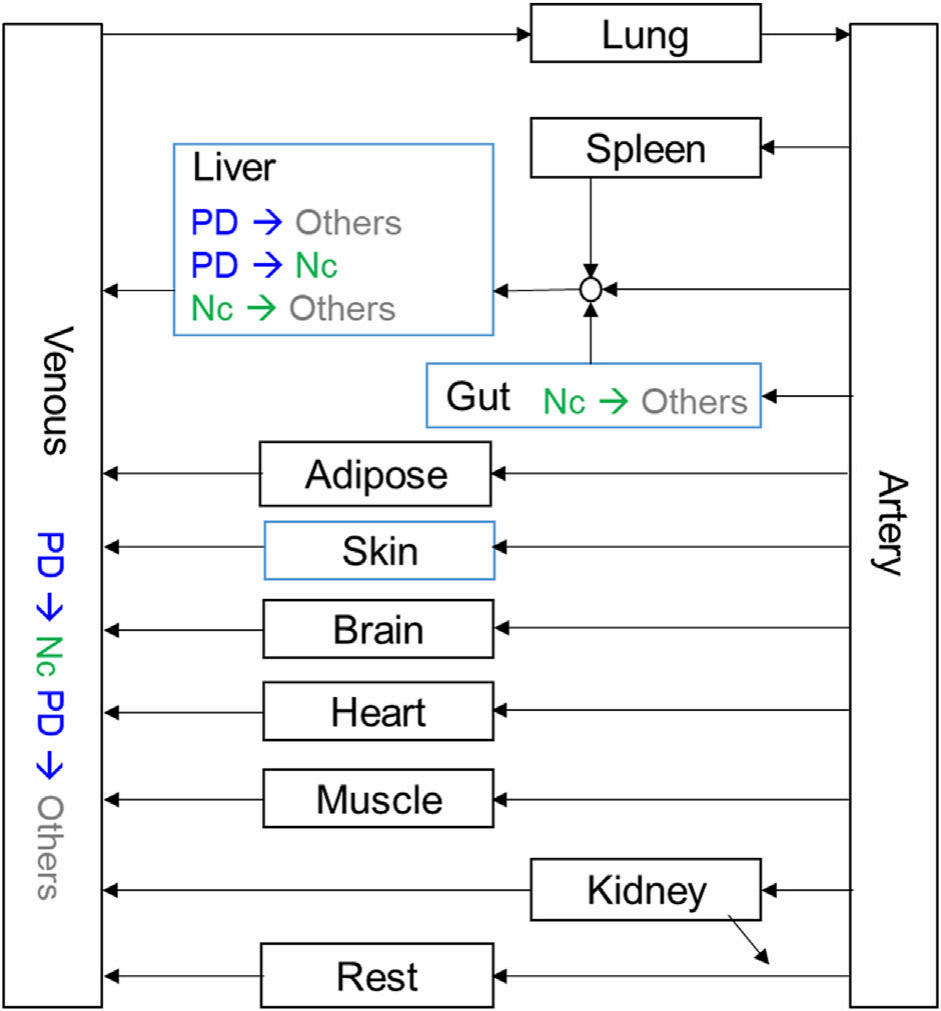
Schematic diagram of the PBPK model. Kinetics for tissues in black boxes were set as perfusion-limited, and kinetics for tissues in blue boxes were set as permeability-limited.

All modeling and simulation were carried out using GastroPlus version 9.5 (Simulations Plus Inc., CA). Physiochemical parameters including blood-to-plasma ratio (R_bp_), pKa, and logP were predicted by ADMET predictor (Simulations Plus Inc., CA). Physiologies of all species, including organ weights, volumes, tissue compositions, and blood perfusion rates, were generated by the program’s internal module. Tissue intracellular unbound fractions were generated by GastroPlus based on predicted or experimentally determined physiochemical and chemical-biological parameters including pKa, logP, plasma unbound fractions (F_up_), R_bp_, solubility, and PAMPA permeability. The PBPK model structure is illustrated in Figure 2 and its parameters are summarized in Supplementary Tables S3–6. The default absorption and dissolution model was applied and effective intestine permeability was predicted by a built-in correlation from the experimental PAMPA permeability result. Parameters including partition coefficients in tissue (Kp), CL_intrinsic_, and permeability surface area product (PStc) were estimated by fitting the model to experimental data using the optimization module in GastroPlus with objective function weights at 1/(y + ŷ)^2^, where y is the observed value and ŷ is the predicted value.

A sensitivity analysis was performed by comparing the normalized sensitivity coefficients (SC) for each input parameter on their effects on plasma AUC or C_max_ by calculating the SC after alternating the best fit parameter values one by one, by a factor of two. SC values for each parameter were determined using the following equation:

$$SC=\left[F\left(p_{1}\right)\;–\;F\left(p_{2}\right)\right]/F\left(p\right)\times p/\left(p_{1}\;–\;p_{2}\right)$$

where p is the initial parameter value; p_1_ and p_2_ are the modified parameter values resulting from a 20% increase or decrease of the initial parameter value; F(p), F(p_1_), and F(p_2_) are the model outputs (C_max_ or AUC) from the input value of p, p_1_, and p_2_, respectively.

## 3 Results

### 3.1 Physiochemical properties of PDN and Nc

The physicochemical properties of PDN and Nc, including pKa, logP and R_bp_, were predicted by *in silico* models based on the chemical structure. Solubility at pH 7.4 was determined experimentally for both compounds. PDN demonstrated a 40-fold increase in solubility compared with Nc. Biological-drug properties including PAMPA permeability, Fu_p_ and R_bp_ were estimated experimentally or from the prediction with software and citation from the published data (Table 2). PDN demonstrates higher unbound fraction in plasma (Fu_p_) than the active compound Nc, with Fu_p_ at around 12%–15%. The results of the positive controls for kinetic solubility and PAMPA permeability are shown in Supplementary Table S7 and Supplementary Table S8.

**TABLE 2:** Physiochemical properties of PDN and Nc.

Parameters	Prodrug (PDN)	Niclosamide (Nc)
**MW**	439	327
**pKa** ^a^	9.58 (acid); 7.80 (base)	10.26 (acid); 8.13 (acid)
**logP** ^a^	3.43	4.08
**Kinetic Solubility (pH 7.4) (mg/mL)**	>0.0435	0.0011
**PAMPA (10** ^**−6**^ ** cm/s)**	>100	<1^b^
**Rbp** ^a^	1.51 (rodent)	1.1 (rodent)
	1.14 (human)	1.60 (human)
**Fu**_**p**_	12.38% (rodent)^a^	0.14% (rodent)^b^
	15.21% (human)	0.16% (human)^b^

^a^ Data predicted by ADMET, predictor v9.0.

^b^ Data from references (Choi et al., 2021; Shamim et al., 2021).

### 3.2 *In vitro* metabolic stability of PDN and Nc in mouse, hamster, and human plasma and liver microsomes

As shown in Table 3, the depletion of Nc in liver microsomes is fast in all three species, with a CL_*in vitro*,app_ ranging from 0.058 to 0.115 mL/min/mg in human and hamster microsomes, respectively. The metabolic stability of Nc in plasma was also investigated, and no depletion was found in plasma from all three species.

**TABLE 3:** Apparent *in vitro* clearance (CL_in vitro,app_) of depletion and biotransformation in plasma and liver microsomes (mean ± SD, *n* = 3).

Tissue	Parameter	Conversion*	Mouse	Hamster	Human
Plasma	k_in vitro,app_ (1/min)	PDN-total	0.0120 ± 0.0015	0.0191 ± 0.0044	0.0025 ± 0.0013
		PDN-Nc	0.00580	0.00453	0.00026
Liver	CL_in vitro,app_ (mL/min/mg)	Nc-total	0.0747 ± 0.0146	0.1152 ± 0.0071	0.0581 ± 0.0153
	K_m_ (nmol/mL)	PDN-total	20.24 ± 3.31	10.82 ± 3.04	33.8 ± 29.1
	V_max_ *_in vitro_*_,app_ (nmol/min/mg)	PDN-total	1.327 ± 0.106	1.238 ± 0.133	0.365 ± 0.187
		PDN-Nc	0.0192	0.0917	0.0080

*PDN-total: Total depletion of PDN; PDN-Nc: Formation of Nc from PDN; Nc-total: Total depletion of Nc.

The *in vitro* metabolic stability of PDN was determined in the plasma and liver fractions of three species. Depletion of PDN was observed in the plasma and the liver microsomes. PDN showed fast biotransformation to Nc in the mouse and hamster plasma, but a much slower biotransformation in the human plasma, as expressed by the reaction rate of k_in vitro,app_. Based on the formation of Nc in plasma, about 51%, 16%, and 10% of depleted PDN were converted to Nc in mouse, hamster, and human plasma, respectively. PDN was also metabolized in the liver microsomes. However, due to the fast metabolism of formed Nc in liver microsomes (sequential metabolism), only a trace amount of Nc was detected after incubation of PDN. Therefore, the metabolism kinetics of PDN was investigated to obtain the V_max,_ _*in vitro*, app_ and K_m_ values of PDN depletion (Table 3). The formation rate parameter of Nc (V_max,_ _*in vitro*,app, PDN-Nc_) was also estimated after PDN was incubated at a high concentration (30 µM) in liver microsomes (Table 3). The result demonstrated that lower than 3% of metabolized PDN was detected to be Nc in mouse and human liver microsomes, while about 10% of PDN was converted to Nc in hamster liver microsomes. In addition, PDN was also incubated in liver cytosolic fractions and the *in vitro* metabolic half-life (t_1/2_) was larger than 120 min, indicating that biotransformation of PDN in liver cytosolic fraction was neglectable.

### 3.3 *In vivo* biotransformation from PDN to Nc and tissue distribution in mice

*In vivo* biotransformation from PDN to Nc and their tissue distribution were studied in mice after IV or PO administration of PDN (Table 4; Table 5). The oral absorption of PDN was high, with bioavailability at 85.6%. The clearance of PDN was moderate with the plasma apparent clearance (CL_p_) of 0.061–0.063 L/h for IV administration of 3 and 10 mg/kg and CL_p_/F of 0.071 L/h for PO administration of 10 mg/kg, respectively. PDN can be efficiently converted to Nc *in vivo* after both IV and PO doses, with the plasma AUC_0-∞_ ratio of Nc to PDN ranging from 0.34 to 0.48. The dose-normalized AUC_0-∞_ of Nc was 216 (h*ng/mL)/(mg/kg) after a PO dose of PDN (10 mg/kg), which was more than 8-fold higher than that reported after a PO dose of Nc (40 mg/kg) (Fan et al., 2019).

**TABLE 4:** Observed and simulated tissue distribution profiles of PDN and formed Nc after IV 3 and 10 mg/kg of PDN in mice. Data were presented as mean (SD).

IV 3 mg/kg												
	**Prodrug (PDN)**						**Niclosamide (Nc)**					
**Tissue**	AUC_0-∞_ (h*ng/mL or h*ng/g)			**t_1/2_ (h)**			AUC_0-∞_ (h*ng/mL or h*ng/g)			**t_1/2_ (h)**		
	**Obs**	**Sim**	**FE**	**Obs**	**Sim**	**FE**	**Obs**	**Sim**	**FE**	**Obs**	**Sim**	**FE**
Adipose	990 (169)	1340	1.35	2.78 (0.73)	1.71	1.62	66 (12)	91	1.38	2.70 (0.67)	3.72	1.38
Brain	1024 (124)	1297	1.27	2.23 (0.35)	1.77	1.26	15 (10)	31	2.12	1.74 (1.11)	3.70	2.13
Heart	5676 (481)	5944	1.05	1.71 (0.23)	1.73	1.01	191 (20)	173	1.10	5.21 (0.82)	3.70	1.41
Intestine	3222 (749)	7353	2.28	2.69 (1.25)	2.02	1.33	5570 (1039)	9107	1.64	2.78 (0.91)	3.91	1.41
Kidney	9833 (1080)	13033	1.33	3.10 (0.23)	1.69	1.83	3587 (485)	4934	1.38	5.76 (4.72)	3.71	1.55
Liver	ND	351	ND	ND	1.79	ND	2423 (288)	4435	1.83	11.61 (0.86)	8.99	1.29
Lung	53071 (4993)	50767	1.05	3.09 (0.25)	1.81	1.71	486 (38)	1055	2.17	3.44 (0.15)	3.70	1.08
Muscle	1828 (221)	3287	1.80	1.91 (0.35)	1.71	1.12	43 (26)	43	1.00	4.00 (3.23)	3.70	1.08
Spleen	16808 (1242)	17262	1.03	3.44 (0.77)	1.67	2.06	183 (113)	179	1.02	4.23 (4.26)	3.70	1.14
Plasma	1193 (96)	1165	1.02	3.07 (0.23)	1.84	1.67	577 (75)	662	1.15	2.66 (0.29)	3.72	1.40
	**CL**_**p**_ **(L/h)**			**Vd**_**ss**_ **(L)**			**AUC ratio (Nc/PDN)**					
Plasma	0.063 (0.01)	0.06	1.00	0.28 (0.03)	0.20	1.41	0.48	0.57	1.18			

IV 10 mg/kg												
	**Prodrug (PDN)**						**Niclosamide (Nc)**					
**Tissue**	**AUC**_**0-∞**_ (h*ng/mL or h*ng/g)			**t**_**1/2**_ **(h)**			**AUC**_**0-∞**_ (h*ng/mL or h*ng/g)			**t_1/2_ (h)**		
	**Obs**	**Sim**	**FE**	**Obs**	**Sim**	**FE**	**Obs**	**Sim**	**FE**	**Obs**	**Sim**	**FE**
Adipose	ND	4509	ND	ND	1.71	ND	260 (30)	372	1.43	4.73 (0.79)	4.17	1.14
Brain	5622 (1331)	4369	1.29	3.39 (1.79)	1.77	1.92	48 (5)	126	2.63	ND	4.15	ND
Heart	31647 (6073)	20013	1.58	2.86 (0.15)	1.73	1.65	455 (107)	711	1.56	5.38 (1.92)	4.15	1.30
Intestine	14586 (1295)	19364	1.33	3.78 (0.00)	2.01	1.88	49903 (18422)	27271	1.83	4.45 (0.32)	4.23	1.05
Kidney	62371 (6713)	43873	1.42	4.78 (0.56)	1.70	2.82	19974 (1492)	20261	1.01	5.12 (0.48)	4.16	1.23
Liver	883 (323)	1186	1.34	3.60 (2.13)	1.79	2.01	8720 (917)	16363	1.88	21.21 (10.38)	9.32	2.28
Lung	173216 (21722)	171029	1.01	3.51 (0.29)	1.81	1.94	2280 (429)	4335	1.90	3.61 (0.35)	4.15	1.15
Muscle	10301 (2999)	11058	1.07	4.65 (2.61)	1.71	2.71	89 (13)	176	1.98	6.28 (5.77)	4.15	1.51
Spleen	63505 (10129)	58080	1.09	3.24 (0.24)	1.67	1.94	454 (142)	736	1.62	3.39 (0.90)	4.15	1.22
Plasma	4095 (497)	3926	1.04	3.52 (0.19)	1.84	1.91	1391 (85)	2723	1.96	4.46 (0.87)	4.17	1.07
	**CL**_**p**_ **(L/h)**			**Vd**_**ss**_ **(L)**			**AUC ratio (Nc/PDN)**					
Plasma	0.061 (0.01)	0.06	1.06	0.31 (0.03)	0.17	1.81	0.34	0.69	2.02			

Note: AUC: area under the curve; t_1/2_: half-life; CL_p_: clearance; Vd_ss_: volume of distribution at steady state; Obs: observed; Sim: simulated; FE: fold of error; AUC_0-∞_ (h*ng/mL or h*ng/g): h*ng/mL for plasma and h*ng/g for other organs and tissues; Parameters for each dosing group were generated from pooled pharmacokinetic profile from 33 mice, 3 mice per time point.

**TABLE 5:** Observed and simulated tissue distribution profiles of PDN and formed Nc after PO 10 mg/kg of PDN in mice. Data were presented as mean (SD).

PO 10 mg/kg															
	**Prodrug (PDN)**														
**Tissue**	AUC_0-∞_ (h*ng/mL or h*ng/g)			**C_max_ (ng/mL or ng/g)**			**t_1/2_ (h)**				**T_max_ (h)**				
	**Obs**	**Sim**	**FE**	**Obs**	**Sim**	**FE**	**Obs**		**Sim**	**FE**	**Obs**	**Sim**		**FE**	
Adipose	2788 (299)	3236	1.16	547 (98)	897	1.64	3.48 (0.24)		2.44	1.43	1.5	1.50		1.05	
Brain	2741 (488)	3559	1.30	715 (84)	1078	1.51	2.45 (0.31)		2.30	1.07	1.5	1.00		1.07	
Heart	10680 (1787)	15882	1.49	3037 (1072)	4804	1.58	2.16 (0.33)		2.30	1.07	1.5	1.00		1.07	
Intestine	37739 (6003)	71330	1.89	15433 (4535)	33878	2.20	1.79 (0.24)		1.38	1.30	0.167	0.167		1.00	
Kidney	40372 (8592)	33988	1.19	5863 (1076)	10257	1.75	2.99 (0.16)		2.31	1.29	1.5	1.00		1.29	
Liver	591 (176)	1346	2.28	240 (109)	483	2.01	2.36 (0.85)		1.91	1.24	1.5	1.00		1.24	
Lung	123875 (23366)	143116	1.16	22559 (463)	43399	1.92	3.29 (0.30)		2.29	1.43	1.5	1.00		1.43	
Muscle	4005 (814)	7939	1.98	1123 (85)	2201	1.96	2.83 (0.55)		2.44	1.16	1.5	1.50		1.29	
Spleen	45737 (9727)	42350	1.08	8077 (519)	12126	1.50	3.16 (0.29)		2.38	1.33	1.5	1.00		1.33	
Plasma	3515 (593)	3358	1.05	606 (185)	1019	1.68	3.19 (0.23)		2.29	1.39	1.5	1.00		1.39	
	**Niclosamide (Nc)**														
Adipose	410 (65)	263	1.56	63 (35)	30	2.11	4.37 (0.48)		4.84	1.11	2	2.00		1.81	
Brain	63 (20)	89	1.42	17 (2)	10	1.63	2.35 (0.91)		4.81	2.05	1.5	2.00		1.02	
Heart	359 (88)	503	1.40	83 (12)	57	1.44	2.75 (0.93)		4.81	1.75	2	2.00		1.14	
Intestine	74369 (23052)	53623	1.39	6197 (1954)	8653	1.40	3.79 (0.33)		3.35	1.13	2	2.00		1.77	
Kidney	8697 (1292)	14316	1.65	1163 (116)	1623	1.40	4.98 (2.68)		4.83	1.03	1.5	2.00		1.94	
Liver	10437 (1267)	13395	1.28	882 (64)	670	1.32	15.78 (5.25)		9.52	1.66	1.5	4.00		2.41	
Lung	1551 (201)	3069	1.98	190 (15)	351	1.84	7.28 (2.58)		4.81	1.51	1.5	2.00		1.32	
Muscle	102 (16)	124	1.22	22 (10)	14	1.52	2.83 (0.61)		4.81	1.70	2	2.00		1.18	
Spleen	439 (190)	521	1.19	94 (12)	59	1.59	3.31 (1.75)		4.81	1.45	1.5	2.00		1.38	
Plasma	1608 (182)	1930	1.20	346 (67)	219	1.58	3.08 (0.26)		4.83	1.57	0.5	2.00		1.28	
	**Prodrug (PDN)**														
	**CL_p_/F (L/h)**			**Vd_ss_/F (L)**			**F (%)**								
Plasma	0.071 (0.01)	0.08	1.14	0.33 (0.05)	0.26	1.25	85.8		80.0	1.07					

Note: AUC: area under the curve; t_1/2_: half-life; C_max_: maximum concentration; T_max_: time to achieve maximum concentration; CL/F: apparent clearance; Vd/F: apparent volume of distribution; F: bioavailability; Obs: observed; Sim: simulated; FE: fold of error; AUC_0-∞_ (h*ng/mL or h*ng/g): h*ng/mL for plasma and h*ng/g for other organs and tissues; Parameters were generated from pooled pharmacokinetic profile from 30 mice, 3 mice per time point.

**FIGURE 3:**
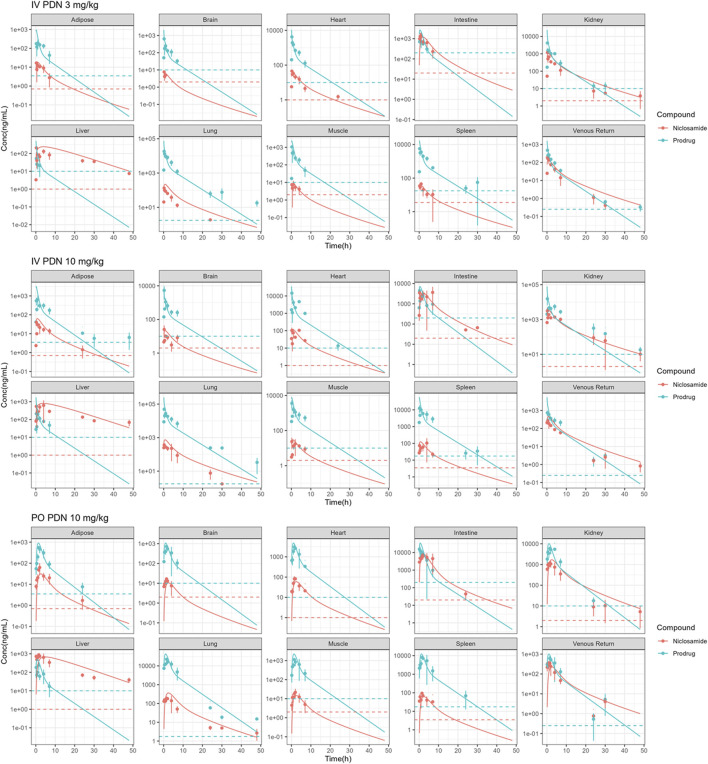
Observed (points) and simulated (solid lines) concentrations of Nc and PDN in plasma, liver, lung, brain, fat, small intestine, heart, kidney, muscle, spleen following IV administration of 3 and 10 mg/kg PDN, and PO administration of 10 mg/kg PDN in mice. (Dotted lines: lowest limits of quantification).

As shown in Table 4 and Table 5, PDN was widely distributed to tissues, with Vd_ss_ or Vd_ss_/F at 0.28–0.33 L in mice. The tissue distribution profile of PDN and Nc was shown in Figure 3. After both IV and PO doses of PDN, the tissue to plasma AUC_0-∞_ ratios in the well-perfused tissues including heart, lung, kidney, liver, spleen, and intestine were high, whereas its distribution to adipose and brain were relatively low. In contrast, the distribution of Nc is very limited. Nc is preferably distributed to the kidney, liver and intestine with high tissue-to-plasma AUC_0-∞_ ratios, while poorly distributed to other tissues with tissue-to-plasma AUC_0-∞_ ratios less than 1. PO administration of 10 mg/kg PDN provided a lung-to-plasma AUC_0-∞_ ratio of Nc at about 0.97, and the lung C_max_ of Nc reached 190 ng/g. After IV administration, PDN presented a terminal t_1/2_ ranging from 3.1–3.5 h in plasma, and similar t_1/2_ in all other tissues (ranging from 1.7–4.8 h). The formed Nc after IV doses of PDN had a terminal t_1/2_ ranging from 2.7–4.5 h in plasma and similar t_1/2_ in other tissues except for the liver. The elimination of Nc in the liver is remarkably 4-fold slower than all other tissue with terminal t_1/2_ ranging from 11.6–21.2 h (Table 4). Similar profiles were observed in the 10 mg/kg PO group (Table 5).

### 3.4 *In vivo* biotransformation from PDN to Nc and the lung concentrations in hamster

**FIGURE 4:**
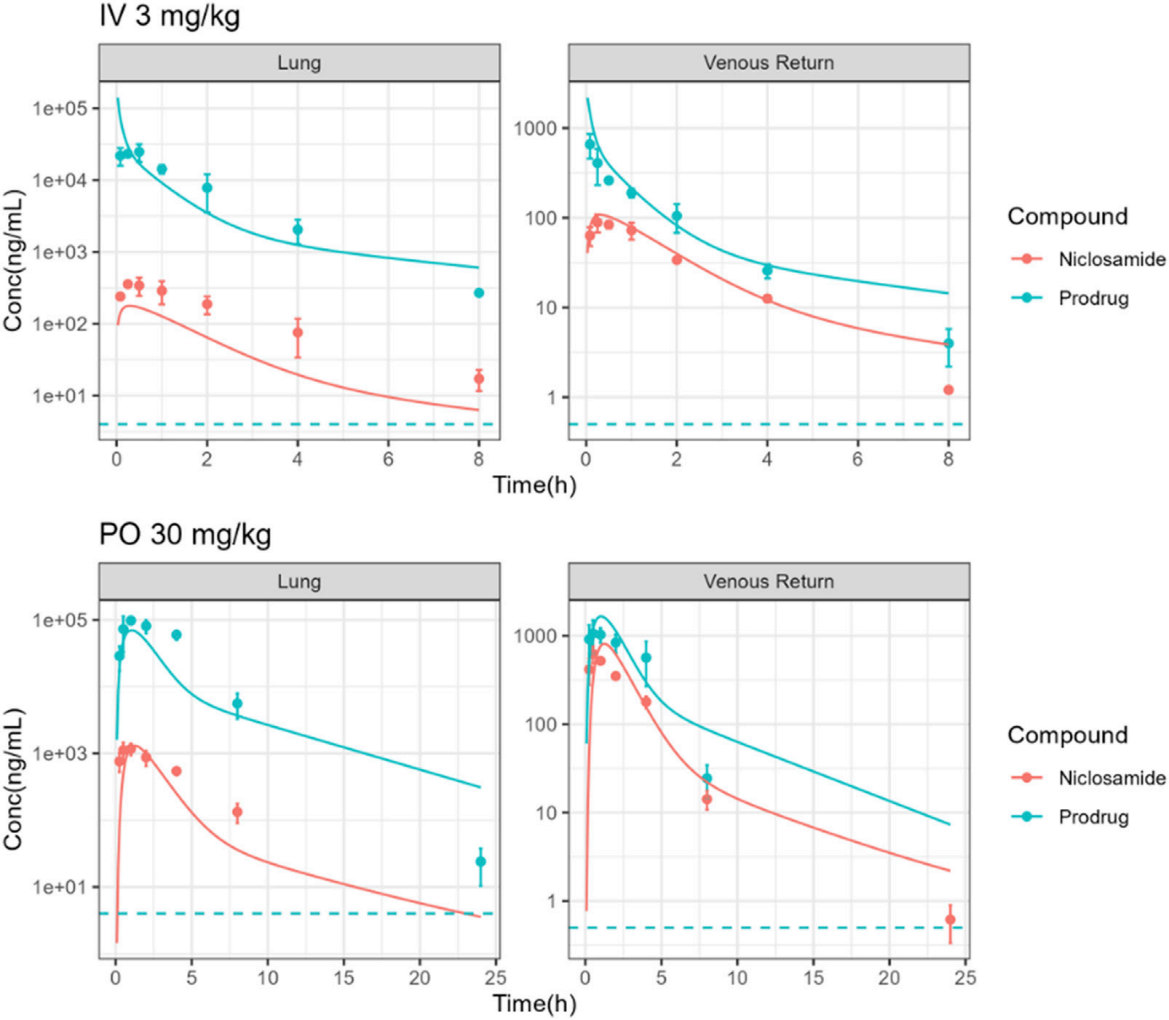
Observed (points) and simulated (solid lines) plasma and lung PK profiles of Nc and PDN following IV administration of 3 mg/kg PDN and PO administration of 30 mg/kg PDN in hamsters. (Dotted lines: lowest limits of detection for both compounds).

*In vivo* biotransformation from PDN to Nc and their lung concentration profile were also studied in hamsters. The plasma and lung concentration-time profiles of PDN and Nc were shown in Figure 4. After 3 mg/kg IV dose of PDN, PDN showed high CL_p_ at 0.48 L/h (Table 6). PDN can be efficiently converted to Nc *in vivo*, with plasma AUC_0-∞_ ratio (Nc/PDN) at 0.32 and 0.44, after both IV and PO doses, very similar to those observed in mice (Table 6; Table 7). The dose normalized plasma AUC_0-∞_ of Nc was 88 (h*ng/mL)/(mg/kg) after PO dose of PDN (30 mg/kg), slightly lower than that observed from the mouse PO dose. The prodrug had a Vd_ss_ at 0.74 L in hamsters (Tables 6). After both IV and PO doses of PDN, the lung to plasma AUC_0-∞_ ratio of PDN is 72 and 107, much higher than those for Nc at 5.0 and 2.6. The lung to plasma AUC_**0-∞**_ratio of Nc observed in hamsters is slightly higher than that in mice, and the lung C_max_ of Nc reached 1170 ng/g after a PO 30 mg/kg dose. Short terminal t_1/2_ values of PDN and Nc were observed at 1.1 and 1.3 h in plasma. The observed t_1/2_ in the lung were similar to those observed in the plasma. After an oral dose of 30 mg/kg, the bioavailability of PDN in hamsters is about 64%, slightly lower than that in mice.

**TABLE 6:** Observed and simulated pharmacokinetic parameters of PDN and Nc in plasma and lung tissues after IV dose of 3 mg/kg PDN in hamsters. Data were presented as mean (SD).

	Prodrug (PDN)					
	AUC_0-∞_ (h*ng/mL or h*ng/g)			**t_1/2_ (h)**		
	**Observed**	**Simulated**	**FE**	**Observed**	**Simulated**	**FE**
Lung	45600 (4266)	37960	1.20	1.1 (0.06)	1.3	1.18
Plasma	631 (42)	863	1.37	1.1 (0.06)	1.4	1.28
	**CL_p_ (L/h)**			**Vd_ss_ (L)**		
	**Observed**	**Simulated**	**FE**	**Observed**	**Simulated**	**FE**
Plasma	0.48 (0.03)	0.35	1.37	0.74 (0.06)	0.69	1.07

	Niclosamide (Nc)					
	AUC_0-∞_ (h*ng/mL or h*ng/g)			**t_1/2_ (h)**		
	**Observed**	**Simulated**	**FE**	**Observed**	**Simulated**	**FE**
Lung	1030 (98)	392	2.61	1.7 (0.13)	1.5	1.13
Plasma	203 (7.7)	240	1.18	1.3 (0.03)	1.5	1.19

Note: AUC: area under the curve; t_1/2_: half-life; C_max_: maximum concentration; T_max_: time to achieve maximum concentration; FE: Fold of error between Observed and Simulated data. AUC_0-∞_ (h*ng/mL or h*ng/g): h*ng/mL for plasma and h*ng/g for lung; Parameters were generated from pooled pharmacokinetic profile from 24 hamsters, 3 hamsters per time point.

**TABLE 7:** Observed and simulated pharmacokinetic parameters of PDN and Nc in plasma and lung tissues after PO dose of 30 mg/kg PDN in hamsters. Data were presented as mean (SD).

Prodrug (PDN)												
**Parameter**	AUC_0-∞_ (h*ng/mL or h*ng/g)			**t_1/2_ (h)**			**T_max_ (h)**			**C_max_ (ng/mL or ng/g)**		
	**Observed**	**Simulated**	**FE**	**Observed**	**Simulated**	**FE**	**Observed**	**Simulated**	**FE**	**Observed**	**Simulated**	**FE**
Lung	462000 (28500)	241000	1.92	2.3 (0.11)	2.4	1.05	1.0	1.0	1.0	98000 (13500)	54500	1.80
Plasma	4320 (559)	5310	1.23	1.8 (0.13)	2.4	1.33	0.5	1.0	2.0	1060 (438)	1300	1.23

	**CL_p_/F (L/h)**			**Vd_ss_/F (L)**										
	**Observed**	**Simulated**	**FE**	**Observed**	**Simulated**	**FE**								
Plasma	0.69 (0.09)	0.57	1.23	1.78 (0.16)	1.92	1.08								

Niclosamide (Nc)												
**Parameter**	AUC_0-∞_ (h*ng/mL or h*ng/g)			**t_1/2_ (h)**			**T_max_ (h)**			**C_max_ (ng/mL or ng/g)**		
	**Observed**	**Simulated**	**FE**	**Observed**	**Simulated**	**FE**	**Observed**	**Simulated**	**FE**	**Observed**	**Simulated**	**FE**
Lung	5020 (294)	7580	1.51	2.4 (0.20)	2.7	1.16	1.0	1.0	1.0	1170 (234)	961	1.22
Plasma	1890 (67.5)	2880	1.52	1.9 (0.05)	2.7	1.47	0.5	1.0	2.0	623 (136)	595	1.05

Note: AUC: area under the curve; t_1/2_: half-life; C_max_: maximum concentration; T_max_: time; FE: Fold of error between Observed and Simulated data. AUC_0-∞_ (h*ng/mL or h*ng/g): h*ng/mL for plasma and h*ng/g for lung; Parameters were generated from pooled pharmacokinetic profile from 21 hamsters, 3 hamsters per time point.

### 3.5 PBPK model

#### 3.5.1 Development of PBPK model depicting mouse pharmacokinetics

The physiochemical and chemical-biological parameters of Nc and PDN used in the PBPK modeling were summarized in Table 2. The PBPK model structure was illustrated in Figure 2. A PBPK model for mice was developed and parametrized based on these available data and the *in vivo* mouse tissue and plasma pharmacokinetic profiles.

The model that described the tissue distribution data was generated from plasma and tissue concentration profiles for PDN and Nc after IV doses of 3 and 10 mg/kg. Perfusion rate-limited kinetics, i.e., assuming that PDN and Nc distributed freely and immediately across the cell membranes and the blood flow rate was the limiting factor for the distribution, was used for all organs except for the liver and skin. According to the distinct tissue concentrations of Nc in the liver, a permeability-limited model was used to restrict the distribution of intracellularly formed Nc across the liver cell membranes, describing the slow elimination t_1/2_ observed (Table 4). Model parameter estimation of all plasma-tissue partition coefficients (Kp) was performed simultaneously using both the IV and PO datasets. Permeability-limited model was applied for the liver and skin compartments for both PDN and Nc. The permeability parameters for these two organs were optimized to match the observed liver and plasma concentration data. The experimentally determined pharmacokinetic profiles were compared with the model simulations. The established PBPK model was able to capture the concentration-time profiles in plasma and various tissues in mice (Figure 3). The pharmacokinetic parameters generated from the PBPK model in mice are summarized in Table 5. The model predictions on key parameters (AUC, t_1/2_, C_max_) are in close agreement (within 2-fold error) with the experimental observations (Table 4; Table 5). A few discrepancies between the experimental data and the model simulations were found after a 10 mg/kg IV dose of PDN in t_1/2_ values for kidney and muscle, where significantly extended t_1/2_ was observed in comparison to the other two dosing groups. Since model parameters were estimated by running simulations for all three experiment groups simultaneously, the simulated t_1/2_ was a compromise of three datasets.

#### 3.5.2 Verification of PBPK model using hamster pharmacokinetics

To validate the PBPK model, the mouse model was applied to another species, i.e., hamster in this study, by incorporating the reported physiologic parameters of hamsters (Mann et al., 1996; Kwon, 2001). The cross-species extrapolation results are shown in Supplementary Table S5 and Supplementary Table S6. Experimental measurements of hamster plasma pharmacokinetics and lung concentrations were used in the model validation. The predicted concentration-time curves were plotted against the observed values (Figure 4). In general, a good agreement was found between experimental and simulated curves and parameters, with the predicted CL_p_/F, Vd_ss_/F, AUC_**0-∞**_, and t_1/2_ within the 2-fold error (FE) range, except for the predicted Nc lung AUC_**0-∞**_ (Table 6; Table 7 **)**.

#### 3.5.3 Human prediction

A human PBPK model was generated with the same structure, using human physiological parameters for a healthy 65-kg subject. The cross-species extrapolation results are shown in Supplementary Table S5 and Supplementary Table S6.

Human prediction was run with a single oral dose of 150, 300, and 600 mg under the fed state. The predicted pharmacokinetic parameters are summarized in Table 8. The predicted human PK profiles show a moderate absorption rate with T_max_ around 3.2 h, low systemic clearance (CL_p_/F), and high volume of distribution (Vd_ss_/F) for PDN. The predicted CL_p_/F in humans after oral dose was significantly lower compared to those observed in mice and hamsters. The simulated formation of Nc was also slower than those observed in preclinical species, demonstrated by the prolonged T_max_ of Nc after the oral dose of PDN (5.1 h in humans vs. 0.5 h in both mice and hamsters). Such a slow formation rate was anticipated based on the lower biotransformation rate from PDN to Nc observed from the *in vitro* metabolism study in both human plasma and liver microsomes. The simulated terminal t_1/2_ of PDN and Nc in humans were therefore also longer than those observed in preclinical species. After oral doses, the plasma t_1/2_ values were ∼3.2 and 3.1 h for PDN and Nc in mice, and were ∼1.8 and 1.9 h in hamsters. However, in humans, the simulated plasma t_1/2_ values were ∼4.9 and 6.3 h for PDN and Nc, respectively (Table 8). Based on the simulations, plasma C_max_ of 0.62, 0.31, and 0.16 μg/mL, and lung C_max_ of 1.75, 0.88, and 0.44 μg/g of Nc were expected after the single dose administration of 600, 300, and 150 mg, respectively (Table 8).

**TABLE 8:** Simulated pharmacokinetic profiles of representative single oral PDN dose at 600, 300, and 150 mg in a 65-kg human subject.

Compound	PDN		Nc	
**Tissue**	**Lung**	**Plasma**	**Lung**	**Plasma**
CL_p_/F (L/h/kg)	NA	2.1	NA	NA
Vd_ss_/F (L/kg)	NA	15	NA	NA
**Dose (mg)**	150			
AUC_0-∞_ (h*µg/mL or h*µg/g)	56.5	1.10	4.03	1.44
t_1/2_ (h)	5.0	4.9	6.2	6.3
C_max_ (μg/mL or μg/g)	7.02	0.14	0.44	0.16
T_max_ (h)	3.2	3.2	5.1	5.1
**Dose (mg)**	300			
AUC_0-∞_ (h*µg/mL or h*µg/g)	113	2.21	8.05	2.86
t_1/2_ (h)	5.0	4.9	6.2	6.3
C_max_ (μg/mL or μg/g)	14.1	0.27	0.88	0.31
T_max_ (h)	3.2	3.2	5.1	5.1
**Dose (mg)**	600			
AUC_0-∞_ (h*µg/mL or h*µg/g)	226.57	4.41	16.05	5.71
t_1/2_ (h)	5.0	4.9	6.2	6.3
C_max_ (μg/mL or μg/g)	28.16	0.55	1.75	0.62
T_max_ (h)	3.2	3.2	5.1	5.1

NA: not applicable; AUC_0-∞_ (h*ng/mL or h*ng/g): h*ng/mL for plasma and h*ng/g for lung.

**FIGURE 5:**
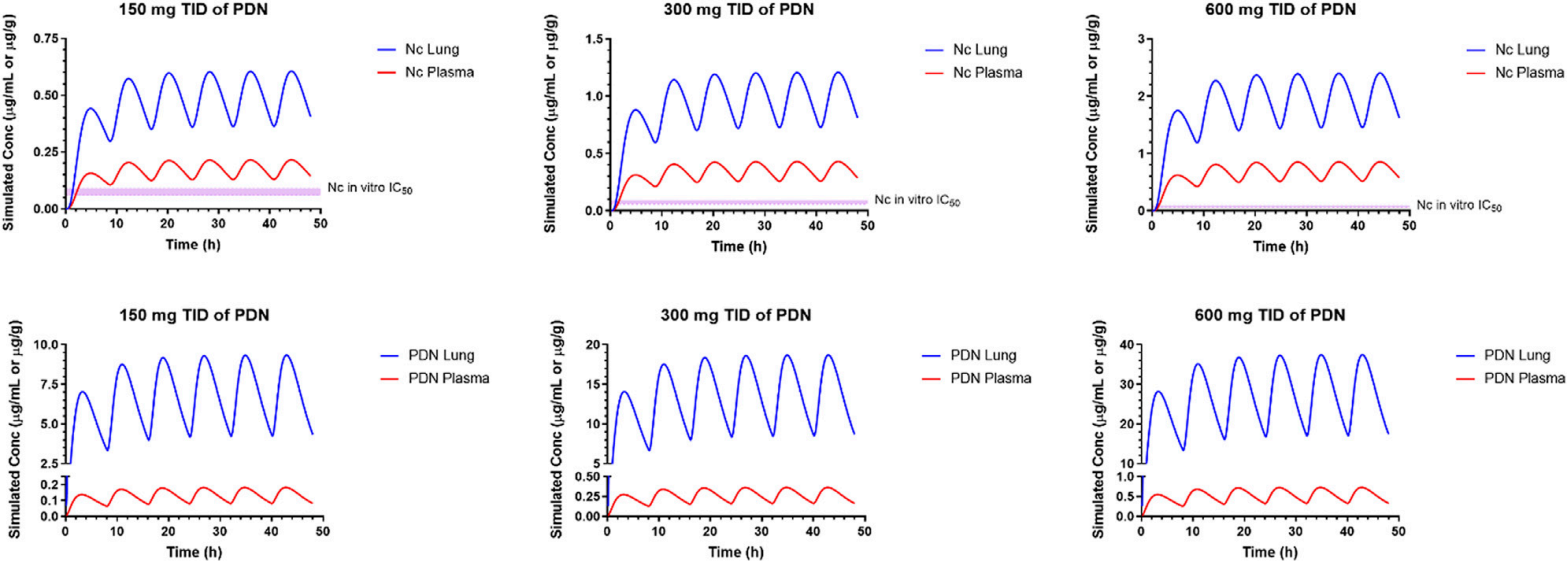
Simulated pharmacokinetic profiles of representative multiple oral PDN dose at 600, 300, and 150 mg, TID in a 65-kg human subject.

According to the simulation results from a single dose and assuming linear kinetics after multiple dose treatment, a TID dose regimen is proposed. In our simulation, after the 150 mg TID doses of PDN for 48 h, the plasma concentrations of Nc at steady-state for C_max_ and C_trough_ were 0.21 and 0.13 μg/mL, and the projected lung concentrations of Nc at steady-state for C_max_ and C_trough_ were 0.60 and 0.36 μg/g, respectively (Figure 5). At a higher dose of TID 600 mg PDN, the steady-state plasma concentrations of Nc were predicted at between 0.85 and 0.56 μg/mL, and the steady-state lung concentrations between 2.40 and 1.46 μg/g (Figure 5).

#### 3.5.4 Sensitivity analysis

**FIGURE 6:**
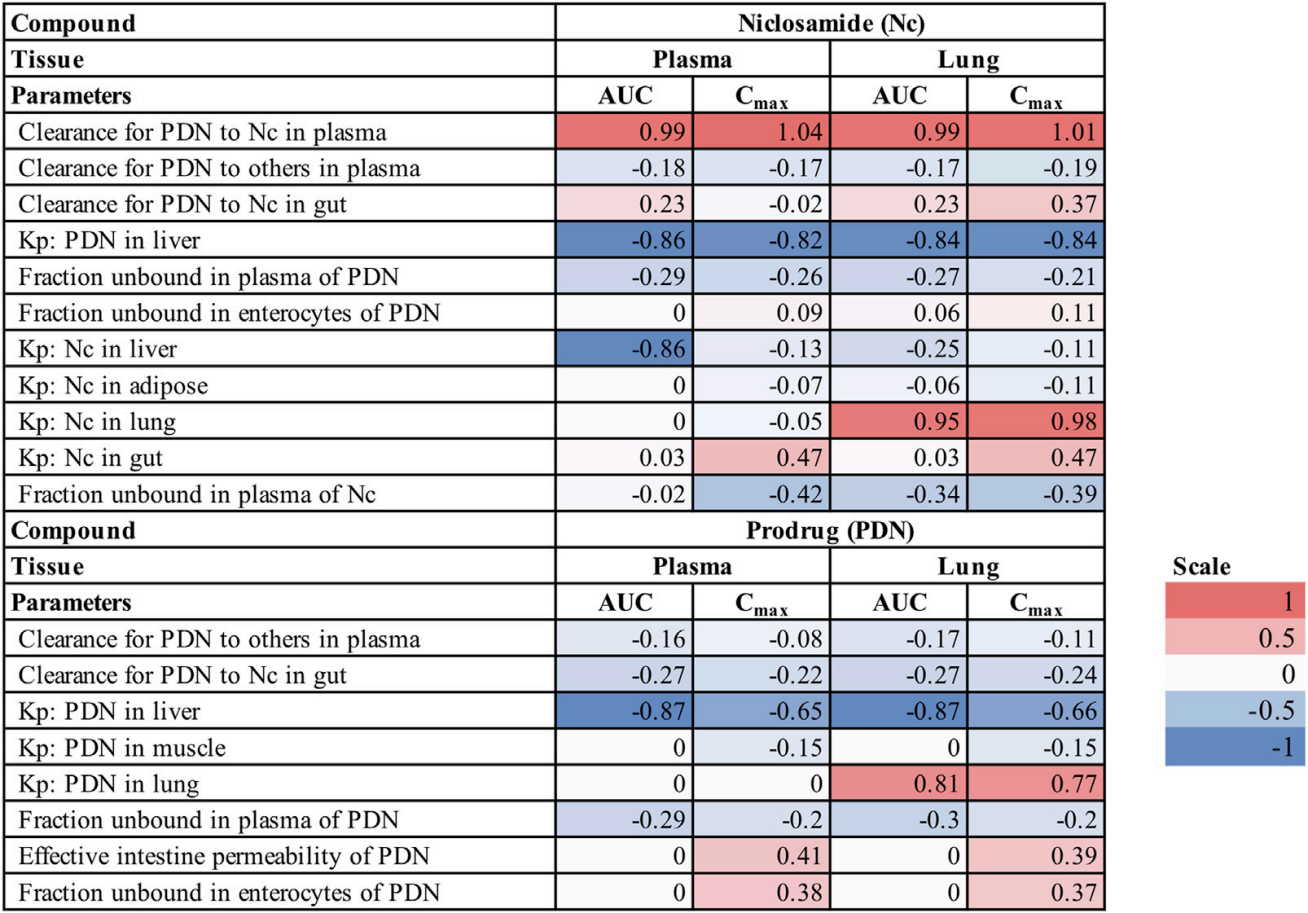
Sensitivity coefficient (SC) of PBPK model parameters for plasma and lung profiles in humans (Only parameters with SC higher than 0.1 or lower than −0.1 are shown).

A sensitivity analysis was performed for the human PBPK model to identify which parameters have the highest influence on the model prediction, focusing on the plasma and lung concentration profiles of Nc and PDN. The normalized sensitivity coefficients (SC) for each PBPK parameter are summarized in Figure 6. Parameters with SC values higher than 0.5 or lower than −0.5 were considered high influencers for the corresponding model output. Most parameters had SC smaller than 0.1, indicating that the model output was very insensitive to the variation of the given parameter.

For concentrations of Nc, the biotransformation rate from PDN to Nc in plasma is the most important influencer for the plasma and lung AUC and C_max_, while the PStc value of PDN in the liver is a second important influencer. The tissue distribution related parameters Kp of Nc have various impacts on the model output of Nc. Kp of Nc to the lung is a determinant for its lung concentrations; PStc to the liver has a large impact on the predicted plasma AUC of Nc, but not on its lung concentrations, and PStc to the gut has a moderate impact on the C_max_ of Nc in both plasma and lung but not on the AUC value. Other parameters including fraction unbound in plasma and enterocytes may also have a minor effect on the pharmacokinetics of Nc. For concentrations of PDN, important parameters were the Kp to liver and Kp to the lung of PDN, with SC greater than 0.5. In addition, the effective intestinal permeability and the fraction unbound in enterocytes of PDN also have a moderate impact on the C_max_ of PDN in both plasma and lung but have little impact on the AUC.

## 4 Discussion

PBPK modeling has been well established for *in vitro* to *in vivo* translation and pharmacokinetic modeling during drug discovery in preclinical species (Bi et al., 2016; Zake et al., 2021) as well as in clinical trials (Lukacova et al., 2016; Miller et al., 2019). With the PBPK model, the dynamics of drug distribution in various tissues can be evaluated and predicted, thus leading to a better understanding of the relationship between target tissue exposure and drug efficacy. In addition, PBPK model is a useful tool to analyze and simulate the ADME process at the organ level by incorporating physiology parameters and mechanisms determined in the *in vitro* model. The use of the PBPK model for human pharmacokinetic predictions is growing and has been considered by regulatory agencies as beneficial to mitigate the uncertainty of a new chemical entity before clinical trials (Wagner et al., 2015; Miller et al., 2019). The present study evaluated a novel prodrug of Nc (PDN) in improving the *in vivo* exposure of Nc in mice and hamsters, and featured a PBPK model that was able to adequately characterize the plasma and organ concentration-time profiles of PDN and Nc. The PBPK model was then applied to simulate the pharmacokinetics of PDN and Nc in humans, supporting the evaluation of its potential clinical use as well as guiding future development strategies for other Nc prodrugs.

We conducted PK experiments in mice and hamsters, the species commonly used for COVID-19 disease model, through IV and PO administration routes and various dosing regimens. We observed a linear pharmacokinetics profile of PDN and formed Nc within the doses studied in both species. A moderate and high CL_p_ in mice and hamsters, and a suitable t_1/2_ were observed for PDN. We also observed extensive tissue distribution and high Vd_ss_ of PDN in both species. PDN was preferably distributed to the highly perfused organs such as the lung, spleen, and kidney, with their AUC in these tissues 8–44 folds higher than in plasma. In contrast, Nc has been reported to have very limited tissue distribution in previous studies (Choi et al., 2021; Shamim et al., 2021). Nc presented high plasma protein binding (>99.8%) and was confined mainly in the plasma with limited tissue distribution after intravenous (IV) injection (Choi et al., 2021; Shamim et al., 2021). The volume of distribution for Nc has been reported to be less than 0.4 L/kg in rats (Choi et al., 2021). After an IV dose of Nc (2 mg/kg) in rats, the concentration of Nc was much higher in the intestine followed by the kidney and liver compared to other tissues including the spleen, lung, and heart (Ye et al., 2015). The tissue concentrations of Nc in mice after oral administration of Nc at 40 mg/kg were also high in the kidney and liver, lower in the lung and heart, and very low in the brain (Tao et al., 2014). Consistent with these previous reports, in our study in mice, Nc showed Kp around or less than 1 in most of the tissues, while much higher partitions were found in intestine, kidney, and liver. In the current study, the dosed PDN was found efficiently converted to Nc in both preclinical species tested, improving the systemic exposure of Nc in mice after oral administration compared to the previously reported concentrations after oral administration of Nc. In our study, after a PO dose of 10 mg/kg PDN, the lung to plasma AUC_0-last_ ratio of Nc was about 0.97, which was 3.6-fold higher than 0.27 as reported after a PO dose of Nc (40 mg/kg) (Fan et al., 2019), and the dose-normalized plasma AUC_0-∞_ of Nc was more than 8-fold higher than that reported after a PO dose of Nc (40 mg/kg) (Fan et al., 2019).

To establish the PBPK model, rather than fully relying on built-in quantitative structure-property relationship data, we obtained critical experimental ADME and physicochemical data. We observed that PDN showed high permeability in artificial lipid membranes in PAMPA measurements. Considering the reported high protein binding of Nc and rat urine pharmacokinetics (Choi et al., 2021; Shamim et al., 2021), Nc is not likely to be excreted *via* urine in its unchanged form, and metabolism would likely be the major elimination route for both PDN and Nc. Therefore, the metabolism of both compounds was studied in liver microsome and plasma to provide mechanistic knowledge for PBPK model building. Metabolism of Nc has been extensively investigated in several previous *in vitro* and *in vivo* studies in mice and humans but has never been investigated in hamsters. In an early phase I study, after a single oral dose of 2000 mg ^14^C-labeled Nc in humans, metabolites were excreted in the form of glucuronides as well as unchanged Nc, 2′,5-dichloro-4′-aminosalicylanilide, and 2′,5-dichloro-4′-acetaminosalicylanilide (Andrews et al., 1982). Recent research also demonstrated that CYPs-mediated biotransformation was not important for the disposition of orally administered Nc, whereas glucuronidation mediated by UGT enzymes significantly contributed to the disposition of Nc (Lu et al., 2016; Fan et al., 2019). The UGT-mediated hepatic CL_*in vitro*,app_ of Nc was reported at 0.18–0.21 mL/min/mg microsomes in mice with apparent K_m_ value determined at 0.09–1.67 μM (Lu et al., 2016; Fan et al., 2019). The mouse hepatic CL_*in vitro*,app_ of Nc in liver microsome determined in our study is 0.075 mL/min/mg microsome, lower than the previously reported values. The discrepancy might be from the different microsomal protein concentrations, Nc concentrations, and different incubation periods. The hepatic CL_*in vitro*,app_ of Nc in human microsome determined in our study is similar to that in mouse microsome, while the CL_*in vitro*,app_ of Nc in human liver microsome was found about ten-fold higher than that in mouse microsomes in a previous study (Lu et al., 2016). Possible reasons for the discrepancy would be that the microsomal protein concentration used for human microsomes is much lower than that for mouse microsomes in the previous study. For compounds with high protein binding, such as Nc, varied microsomal protein concentrations may alter its unbound concentration in the incubation system, resulting in varied apparent CL_*in vitro*,app_ (Obach, 1999). Unlike the previous study, our *in vitro* system used the same microsome protein concentration for different species and could give a better cross-species prediction for the hepatic CL_*in vitro*,app_ of Nc. We also determined the hamster hepatic CL_*in vitro*,app_ of Nc, which was found slightly higher than the mouse hepatic CL_*in vitro*,app_ of Nc. Consistent with the reported stability of Nc towards human and rat plasma carboxylesterases (Bradshaw et al., 2018), no depletion of Nc was found in plasma from all three species tested in our study. Biotransformation from PDN to Nc was also explored. PDN showed fast biotransformation to Nc in the mouse and hamster plasma, but a much lower rate in the human plasma. In mouse and human liver microsomes, only a small proportion of PDN was converted to Nc, while slightly higher proportion of PDN was converted to Nc in hamster liver microsomes. Since a distinct concentration-time curve of Nc was observed in the liver compared to other tissues, the saturable metabolic kinetics of PDN was estimated in liver microsomes and embedded in the PBPK model. Biotransformation of PDN in liver cytosolic fraction was also tested but found neglectable. The plasma protein binding of PDN in rodent plasma could not be experimentally determined due to its instability after 1 h incubation at 37°C and was estimated by an *in silico* model. Since the Fu_p_ determined in human plasma experimentally was very close to that predicted by the *in silico* model (15.2% vs. 15.8%), the predicted rodent Fu_p_ was adapted for the PBPK modeling.

*In vitro*-*in vivo* extrapolation was applied to fit our *in vitro* data to the PBPK model for the simulation of *in vivo* pharmacokinetic profiles in mice. It has been widely reported that the liver microsomal incubation system tends to underpredict hepatic *in vivo* CL_intrinsic,_ especially for drugs with high plasma protein binding such as Nc or high *in vivo* CL_intrinsic_ such as PDN. The underprediction could be attributed to factors such as the *in vitro* non-specific binding and the lability of enzymes and transporters during preparation and storage processes. Application of the empirical scaling factor (ESF) was reported to successfully correct for such discrepancy (Obach, 1999; Wood et al., 2017; Hallifax and Houston, 2019). The estimated ESF also reflects the contribution of each biotransformation process at the organism level and facilitates more specific and accurate cross-species extrapolation based on the *in vitro* findings. It is worth noting that, Nc undergoes first-pass metabolism by the intestine and liver. *In vitro* stability of Nc showed that Nc-glucuronide formation in intestinal microsomes was about 10-fold greater than that in the liver (Fan et al., 2019). A recent report of *in vitro* assays identified UGT1A1 as the most active human enzyme for the glucuronidation of Nc (Lu et al., 2016), which is highly expressed in both liver and small intestine (Uhlen et al., 2015; Proteinatlas, 2021). Similarly, hydrolysis enzymes such as carboxylesterases and paraoxonase that may contribute to the biotransformation from PDN to Nc in the liver are also expressed in the intestine (Yang et al., 2011). Therefore, in our PBPK model, intestinal clearance of Nc and intestinal biotransformation from PDN to Nc were incorporated. In our study, we did not conduct *in vitro* metabolism study in intestinal microsomes because hamster intestinal microsomes were not available. In this case, following the common practice to estimate *in vivo* intestinal metabolic rates, the metabolic kinetic parameters determined in liver microsomes were scaled to the *in vivo* intestinal biotransformation rates in our PBPK model. *In vitro*-*in vivo* extrapolation was applied to estimate the absorption rate of PDN in three species by using the built-in correlation of GastroPlus. The high PAMPA permeability of PDN projected a complete absorption (>99%) into enterocytes in mice, hamsters, and humans.

The established PBPK model was then refined and verified using concentration-time profiles in various tissues and plasma from two preclinical species. The simulated concentration profiles in various tissues and generated pharmacokinetic parameters in mice are within the acceptance criterion (2-fold error range of observed data). The PBPK model optimized for mice was also able to provide an acceptable prediction of plasma and lung concentrations in the hamsters after PO administration of PDN, providing predictability for the simulation results in humans. The simulated absorption of PDN in humans indicated a complete but slightly slower oral absorption compared to hamsters and mice. Low clearance and high volume of distribution leading to a longer t_1/2_ of PDN in humans were predicted. The formation of Nc was also predicted to be much slower than that in hamsters and mice. After the oral dose of PDN, the overall Nc to PDN ratio predicted in humans (AUC_0-∞_ ratio Nc/PDN of 1.3 after PO 150 mg for a human subject or 2.3 mg/kg dose) was higher than those observed in hamsters (0.44 after PO 30 mg/kg dose) and mice (0.46 after PO 10 mg/kg), possibly due to the slower elimination of Nc in human. Accordingly, after the oral dose of PDN, the t_1/2_ predicted for Nc was also longer in humans (6.3 h) than those observed in rodents (3.1 h in mice and 1.9 in hamsters). In our simulations for humans, after the TID 150 mg doses of PDN, the steady-state plasma concentrations of Nc were at 130–210 ng/mL. Such PK profile is comparable to those observed in phase I clinical trial of Nc in prostate cancer patients, where the plasma C_max_ after TID 500 mg oral Nc dose ranged from 35.7 to 182 ng/mL (Schweizer et al., 2018). At a higher dose of TID 600 mg PDN, the simulated steady-state plasma concentrations of Nc were 0.56–0.85 μg/mL, higher than the observed Nc concentrations from a phase II clinical trial where median plasma C_max_ of 0.665 μg/mL was achieved after 2 g QD oral Nc dose (Burock et al., 2018). Our model was also able to predict the lung concentrations of Nc in humans. After the TID 600 mg doses of PDN, steady-state lung concentrations of Nc were 1.46–2.40 μg/g tissue, which are 16–120 folds higher than the IC_50_ of Nc in the *in vitro* model (20–91 ng/mL) against SARS-CoV-2 (Weiss et al., 2021).

The sensitivity and uncertainty of the model were also systematically assessed. In the human PBPK model, the biotransformation rate from PDN to Nc in plasma is the most important influencer, while the PStc value of PDN to the liver is a second important influencer for Nc plasma and lung exposure. In contrast, the biotransformation rate from PDN to Nc in the liver has little effect on altering the Nc systemic exposure. This could be due to the low membrane permeability of Nc manifested by the significantly prolonged elimination of Nc in mouse liver. The PStc value of Nc in the liver is also a significant influencer for Nc plasma exposure. Based on this analysis, future development of Nc prodrugs should put more emphasis on the plasma biotransformation rate. Fu_p_ of Nc also has some impact on the plasma and lung concentration profiles of Nc. For high protein binding compounds like Nc, the experimental variation of the unbound fraction value could be very large due to the limited detection sensitivity. Considering such large variations on Fu_p_ of Nc, Fu_p_ could be one of the reasons for the uncertainty of the current PBPK model.

A limitation of the current model is the mixed metabolic kinetics. Linear biotransformation rate of PDN was assumed in plasma. In our preclinical studies, CL_p_ of PDN is consistent, and plasma and tissue AUC_0-∞_ of PDN and Nc are proportional to the dose. Linear pharmacokinetics of Nc and PDN was observed when the plasma concentrations are below 623 and 1063 ng/mL for Nc and PDN, and liver concentrations are below 822 and 240 ng/g for Nc and PDN. The current PBPK model may overestimate the elimination of PDN or biotransformation from PDN to Nc in plasma and give unreliable predictions when the dose increases, resulting in higher plasma or liver concentrations. Variability in biotransformation may also come from individual differences in the expression or function of related enzymes. Therefore, further studies on identifying the metabolic enzymes involved are warranted to improve the model prediction accuracy in different situations.

Due to its inherently low bioavailability and low systemic exposure, orally dosed Nc is well-tolerated. The maximum approved oral dose of Nc in its anthelmintic application is 2 g QD (Burock et al., 2018). The acute toxicity test in rats showed its oral LD_50_ > 5000 mg/kg (Andrews et al., 1982). Dose-limiting toxicities of Nc are mainly gastro-intestinal tract related, such as nausea, vomiting, diarrhea, and colitis (Schweizer et al., 2018). However, the maximum tolerated dose of Nc has never been determined by IV administration. By applying the prodrug strategies, much higher amounts of Nc were delivered into the systemic circulation. Based on our current finding, high exposure levels of Nc were observed in the liver, intestine and kidney. The potential risk of high tissue exposure levels of Nc after the intake of prodrug needs to be determined in future toxicity and safety studies.

## 5 Conclusion

In this study, we evaluated *in vitro* ADME properties and pharmacokinetics of a novel prodrug of Nc (PDN; NCATS-SM4705), which was designed to improve the *in vivo* exposure of Nc. PDN was efficiently converted to Nc in both liver and blood, resulting in an improved systemic exposure of Nc in mice and hamsters after oral administration. A PBPK model was developed to adequately simulated the pharmacokinetic and tissue distribution profiles of PDN and the formed Nc in mouse and hamster, and the analysis of this model indicated that the plasma and lung concentration of Nc is most sensitive to the permeability of PDN and Nc across the hepatocyte membranes, as well as the biotransformation rate from prodrug to Nc in plasma. Our predicted Nc concentrations in human plasma and lung suggest that a TID dose of 300 mg PDN would provide a lung concentration of Nc 8- to 60-fold higher than the IC_50_ against SARS-CoV-2 reported from *in vitro* cell assays.

## Data Availability

The original contributions presented in the study are included in the article/Supplementary Material, further inquiries can be directed to the corresponding author.

## References

[B1] Andrews P. Thyssen J. Lorke D. (1982). The biology and toxicology of molluscicides, Bayluscide. Bayluscide. Pharmacol. Ther. 19 (2), 245–295. 10.1016/0163-7258(82)90064-x 6763710

[B2] Backer V. Sjobring U. Sonne J. Weiss A. Hostrup M. Johansen H. K. (2021). A randomized, double-blind, placebo-controlled phase 1 trial of inhaled and intranasal niclosamide: A broad spectrum antiviral candidate for treatment of COVID-19. Lancet Reg. Health Eur. 4, 100084. 10.1016/j.lanepe.2021.100084 33842908 PMC8021896

[B3] Bi Y. Deng J. Murry D. J. An G. (2016). A whole-body physiologically based pharmacokinetic model of gefitinib in mice and scale-up to humans. AAPS J. 18 (1), 228–238. 10.1208/s12248-015-9836-3 26559435 PMC4706283

[B4] Bradshaw P. R. Wilson I. D. Gill R. U. Butler P. J. Dilworth C. Athersuch T. J. (2018). Metabolic hydrolysis of aromatic amides in selected rat, minipig, and human *in vitro* systems. Sci. Rep. 8 (1), 2405. 10.1038/s41598-018-20464-4 29402925 PMC5799297

[B5] Burock S. Daum S. Tröger H. Kim T. D. Krüger S. Rieke D. T. (2018). Niclosamide a new chemotherapy agent? Pharmacokinetics of the potential anticancer drug in a patient cohort of the NIKOLO trial. Am. Soc. Clin. Oncol. 36, e14536. 10.1200/JCO.2018.36.15_suppl.e14536

[B6] Cairns D. M. Dulko D. Griffiths J. K. Golan Y. Cohen T. Trinquart L. (2022). Efficacy of niclosamide vs placebo in SARS-CoV-2 respiratory viral clearance, viral shedding, and duration of symptoms among patients with mild to moderate COVID-19: A phase 2 randomized clinical trial. JAMA Netw. Open 5 (2), e2144942. 10.1001/jamanetworkopen.2021.44942 35138402 PMC8829666

[B7] Choi H. I. Kim T. Lee S. W. Woo Kim J. Ju Noh Y. Kim G. Y. (2021). Bioanalysis of niclosamide in plasma using liquid chromatography-tandem mass and application to pharmacokinetics in rats and dogs. J. Chromatogr. B Anal. Technol. Biomed. Life Sci. 1179, 122862. 10.1016/j.jchromb.2021.122862 PMC8286234 34332199

[B8] Elkihel L. Loiseau P. M. Bourass J. Gayral P. Letourneux Y. (1994). Synthesis and orally macrofilaricidal evaluation of niclosamide lymphotropic prodrugs. Arzneimittelforschung 44 (11), 1259–1264. 7848342

[B9] Fan X. Li H. Ding X. Zhang Q. Y. (2019). Contributions of hepatic and intestinal metabolism to the disposition of niclosamide, a repurposed drug with poor bioavailability. Drug Metab. Dispos. 47 (7), 756–763. 10.1124/dmd.119.086678 31040114 PMC6592404

[B10] Hallifax D. Houston J. B. (2019). Use of segregated hepatocyte scaling factors and cross-species relationships to resolve clearance dependence in the prediction of human hepatic clearance. Drug Metab. Dispos. 47 (3), 320–327. 10.1124/dmd.118.085191 30610004

[B11] Huang W. Shamim K. Tang H. (2022). Small molecule inhibitors of SARS-COV-2 infections. Geneva: World Intellectual Property Organization. WO 2022/109148.

[B12] Jara M. O. Warnken Z. N. Sahakijpijarn S. Moon C. Maier E. Y. Christensen D. J. (2021). Niclosamide inhalation powder made by thin-film freezing: Multi-dose tolerability and exposure in rats and pharmacokinetics in hamsters. Int. J. Pharm. 603, 120701. 10.1016/j.ijpharm.2021.120701 33989748 PMC8112893

[B13] Jeon S. Ko M. Lee J. Choi I. Byun S. Y. Park S. (2020). Identification of antiviral drug candidates against SARS-CoV-2 from FDA-approved drugs. Antimicrob. Agents Chemother. 64 (7), e00819–e00820. 10.1128/AAC.00819-20 32366720 PMC7318052

[B14] Kuepfer L. Niederalt C. Wendl T. Schlender J. F. Willmann S. Lippert J. (2016). Applied concepts in PBPK modeling: How to build a PBPK/PD model. CPT Pharmacometrics Syst. Pharmacol. 5 (10), 516–531. 10.1002/psp4.12134 27653238 PMC5080648

[B15] Kwon Y. (2001). Handbook of essential pharmacokinetics, pharmacodynamics and drug metabolism for industrial scientists. Berlin: Springer Science & Business Media.

[B16] Lu D. Ma Z. Zhang T. Zhang X. Wu B. (2016). Metabolism of the anthelmintic drug niclosamide by cytochrome P450 enzymes and UDP-glucuronosyltransferases: Metabolite elucidation and main contributions from CYP1A2 and UGT1A1. Xenobiotica 46 (1), 1–13. 10.3109/00498254.2015.1047812 26068521

[B17] Lukacova V. Goelzer P. Reddy M. Greig G. Reigner B. Parrott N. (2016). A physiologically based pharmacokinetic model for ganciclovir and its prodrug valganciclovir in adults and children. AAPS J. 18 (6), 1453–1463. 10.1208/s12248-016-9956-4 27450227

[B18] Mann S. Droz P. O. Vahter M. (1996). A physiologically based pharmacokinetic model for arsenic exposure. I. Development in hamsters and rabbits. Toxicol. Appl. Pharmacol. 137 (1), 8–22. 10.1006/taap.1996.0052 8607145

[B19] Miller N. A. Reddy M. B. Heikkinen A. T. Lukacova V. Parrott N. (2019). Physiologically based pharmacokinetic modelling for first-in-human predictions: An updated model building strategy illustrated with challenging industry case studies. Clin. Pharmacokinet. 58 (6), 727–746. 10.1007/s40262-019-00741-9 30729397

[B20] Obach R. S. (1999). Prediction of human clearance of twenty-nine drugs from hepatic microsomal intrinsic clearance data: An examination of *in vitro* half-life approach and nonspecific binding to microsomes. Drug Metab. Dispos. 27 (11), 1350–1359. 10534321

[B21] Parikh M. Liu C. Wu C. Y. Evans C. P. Dall'Era M. Robles D. (2021). Phase Ib trial of reformulated niclosamide with abiraterone/prednisone in men with castration-resistant prostate cancer. Sci. Rep. 11 (1), 6377. 10.1038/s41598-021-85969-x 33737681 PMC7973745

[B22] Proteinatlas (2021). Human protein atlas. [updated 2021-02-24. Available at: http://www.proteinatlas.org (Accessed 02 24, 2021).

[B23] Reddy G. B. Kerr D. L. Spasojevic I. Tovmasyan A. Hsu D. S. Brigman B. E. (2020). Preclinical testing of a novel niclosamide stearate prodrug therapeutic (NSPT) shows efficacy against osteosarcoma. Mol. Cancer Ther. 19 (7), 1448–1461. 10.1158/1535-7163.MCT-19-0689 32371588

[B24] Schweizer M. T. Haugk K. McKiernan J. S. Gulati R. Cheng H. H. Maes J. L. (2018). A phase I study of niclosamide in combination with enzalutamide in men with castration-resistant prostate cancer. PLoS One 13 (6), e0198389. 10.1371/journal.pone.0198389 29856824 PMC5983471

[B25] Shah P. Kerns E. Nguyen D. T. Obach R. S. Wang A. Q. Zakharov A. (2016). An automated high-throughput metabolic stability assay using an integrated high-resolution accurate mass method and automated data analysis software. Drug Metab. Dispos. 44 (10), 1653–1661. 10.1124/dmd.116.072017 27417180 PMC5034701

[B26] Shamim K. Xu M. Hu X. Lee E. M. Lu X. Huang R. (2021). Application of niclosamide and analogs as small molecule inhibitors of Zika virus and SARS-CoV-2 infection. Bioorg Med. Chem. Lett. 40, 127906. 10.1016/j.bmcl.2021.127906 33689873 PMC7936759

[B27] Sun H. Nguyen K. Kerns E. Yan Z. Yu K. R. Shah P. (2017). Highly predictive and interpretable models for PAMPA permeability. Bioorg Med. Chem. 25 (3), 1266–1276. 10.1016/j.bmc.2016.12.049 28082071 PMC5291813

[B28] Sun H. Shah P. Nguyen K. Yu K. R. Kerns E. Kabir M. (2019). Predictive models of aqueous solubility of organic compounds built on A large dataset of high integrity. Bioorg Med. Chem. 27 (14), 3110–3114. 10.1016/j.bmc.2019.05.037 31176566 PMC8274818

[B29] Tao H. Zhang Y. Zeng X. Shulman G. I. Jin S. (2014). Niclosamide ethanolamine-induced mild mitochondrial uncoupling improves diabetic symptoms in mice. Nat. Med. 20 (11), 1263–1269. 10.1038/nm.3699 25282357 PMC4299950

[B30] Uhlen M. Fagerberg L. Hallstrom B. M. Lindskog C. Oksvold P. Mardinoglu A. (2015). Proteomics. Tissue-based map of the human proteome. Science 347 (6220), 1260419. 10.1126/science.1260419 25613900

[B31] Wagner C. Zhao P. Pan Y. Hsu V. Grillo J. Huang S. M. (2015). Application of physiologically based pharmacokinetic (PBPK) modeling to support dose selection: Report of an FDA public workshop on PBPK. CPT Pharmacometrics Syst. Pharmacol. 4 (4), 226–230. 10.1002/psp4.33 26225246 PMC4429576

[B32] Weiss A. Touret F. Baronti C. Gilles M. Hoen B. Nougairede A. (2021). Niclosamide shows strong antiviral activity in a human airway model of SARS-CoV-2 infection and a conserved potency against the Alpha (B.1.1.7), Beta (B.1.351) and Delta variant (B.1.617.2). PLoS One 16 (12), e0260958. 10.1371/journal.pone.0260958 34855904 PMC8639074

[B33] Wood F. L. Houston J. B. Hallifax D. (2017). Clearance prediction methodology needs fundamental improvement: Trends common to rat and human hepatocytes/microsomes and implications for experimental methodology. Drug Metab. Dispos. 45 (11), 1178–1188. 10.1124/dmd.117.077040 28887366

[B34] Xu J. Shi P. Y. Li H. Zhou J. (2020). Broad spectrum antiviral agent niclosamide and its therapeutic potential. ACS Infect. Dis. 6 (5), 909–915. 10.1021/acsinfecdis.0c00052 32125140 PMC7098069

[B35] Yang Y. Aloysius H. Inoyama D. Chen Y. Hu L. (2011). Enzyme-mediated hydrolytic activation of prodrugs. Acta Pharm. Sin. B 1 (3), 143–159. 10.1016/j.apsb.2011.08.001

[B36] Ye Y. Zhang X. Zhang T. Wang H. Wu B. (2015). Design and evaluation of injectable niclosamide nanocrystals prepared by wet media milling technique. Drug Dev. Ind. Pharm. 41 (9), 1416–1424. 10.3109/03639045.2014.954585 25204767

[B37] Zake D. M. Kurlovics J. Zaharenko L. Komasilovs V. Klovins J. Stalidzans E. (2021). Physiologically based metformin pharmacokinetics model of mice and scale-up to humans for the estimation of concentrations in various tissues. PLoS One 16 (4), e0249594. 10.1371/journal.pone.0249594 33826656 PMC8026019

