# Supplementary material for: Use of physiological based pharmacokinetic modeling for cross-species prediction of pharmacokinetic and tissue distribution profiles of a novel niclosamide prodrug
Source: Front Pharmacol. 2023 Apr 11;14:1099425. doi: 10.3389/fphar.2023.1099425 (PMC10126473; doi:10.3389/fphar.2023.1099425)
Supplement: Supplementary file 1 [file Table1.DOCX]

**Supplementary materials**

**Supplementary Table 1. Linear range, precision and accuracy of the bioanalysis method for PDN in various tissues and plasma (n=4-6)**

| **Tissue** | **Nominal Conc. (ng/mL)** | **Precision**  **(%)** | **Accuracy**  **(%)** | **Linear Range (ng/mL)** | **Dilution Factor** |
| --- | --- | --- | --- | --- | --- |
| **Lung** | 1 | 7.7 | 114.0 | 1-10000 | 4 |
|  | 10 | 8.5 | 98.7 |  |  |
|  | 100 | 6.1 | 97.8 |  |  |
|  | 1000 | 2.9 | 106.0 |  |  |
|  | 10000 | 3.4 | 107.5 |  |  |
| **Plasma** | 1 | 13.2 | 112.6 | 1-1000 | 1 |
|  | 10 | 14.4 | 99.3 |  |  |
|  | 100 | 15.0 | 101.4 |  |  |
|  | 1000 | 5.9 | 111.2 |  |  |
| **Muscle** | 10 | 11.3 | 88.3 | 5-1000 | 4 |
|  | 100 | 6.8 | 102.1 |  |  |
|  | 1000 | 8.5 | 103.4 |  |  |
| **Liver** | 10 | 11.1 | 92.2 | 5-1000 | 4 |
|  | 100 | 8.1 | 100.7 |  |  |
|  | 1000 | 4.5 | 105.1 |  |  |
| **Kidney** | 10 | 7.7 | 86.0 | 5-1000 | 4 |
|  | 100 | 4.5 | 101.8 |  |  |
|  | 1000 | 6.2 | 102.6 |  |  |
| **Adipose** | 10 | 13.5 | 105.5 | 5-1000 | 1.4 |
|  | 100 | 7.5 | 113.8 |  |  |
|  | 1000 | 7.9 | 110.6 |  |  |
| **Spleen** | 10 | 9.4 | 101.8 | 5-10000 | 7 |
|  | 100 | 6.9 | 104.8 |  |  |
|  | 1000 | 5.0 | 115.6 |  |  |
|  | 10000 | 7.7 | 108.2 |  |  |
| **Heart** | 10 | 5.6 | 112.0 | 5-1000 | 4 |
|  | 100 | 3.0 | 115.8 |  |  |
|  | 1000 | 4.3 | 115.5 |  |  |
| **Small Intestine** | 50 | 3.6 | 97.0 | 20-1000 | 20 |
|  | 100 | 7.4 | 90.9 |  |  |
|  | 1000 | 9.7 | 96.7 |  |  |
| **Brain** | 10 | 5.7 | 101.5 | 5-1000 | 4 |
|  | 100 | 4.2 | 99.3 |  |  |
|  | 1000 | 4.4 | 98.2 |  |  |

**Supplementary Table 2. Linear range, precision and accuracy of the bioanalysis method for Nc in plasma and various tissues (n=4-6)**

| **Tissue** | **Nominal Conc. (ng/mL)** | **Precision**  **(%)** | **Accuracy**  **(%)** | **Linear Range (ng/mL)** | **Dilution Factor** |
| --- | --- | --- | --- | --- | --- |
| **Plasma** | 1 | 17.3 | 97.6 | 1-1000 | 1 |
|  | 10 | 2.2 | 103.8 |  |  |
|  | 100 | 5.9 | 100.3 |  |  |
|  | 1000 | 5.1 | 87.9 |  |  |
| **Lung** | 1 | 18.2 | 100.6 | 1-1000 | 4 |
|  | 10 | 4.5 | 103.0 |  |  |
|  | 100 | 3.3 | 108.5 |  |  |
|  | 1000 | 3.0 | 108.0 |  |  |
| **Muscle** | 1 | 10.5 | 101.8 | 1-1000 | 4 |
|  | 10 | 9.1 | 86.3 |  |  |
|  | 100 | 6.9 | 91.9 |  |  |
|  | 1000 | 5.8 | 87.1 |  |  |
| **Liver** | 1 | 11.0 | 116.8 | 1-1000 | 4 |
|  | 10 | 7.7 | 100.8 |  |  |
|  | 100 | 5.4 | 99.8 |  |  |
|  | 1000 | 3.5 | 97.9 |  |  |
| **Kidney** | 1 | 20 | 92 | 1-1000 | 4 |
|  | 10 | 10.6 | 104.5 |  |  |
|  | 100 | 6.2 | 113.0 |  |  |
|  | 1000 | 5.9 | 106.6 |  |  |
| **Adipose** | 1 | 8.5 | 93.2 | 1-1000 | 1.4 |
|  | 10 | 11.8 | 95.1 |  |  |
|  | 100 | 6.8 | 105.5 |  |  |
|  | 1000 | 9.4 | 100.2 |  |  |
| **Spleen** | 1 | 16.5 | 93.3 | 1-1000 | 7 |
|  | 10 | 8.5 | 92.3 |  |  |
|  | 100 | 4.6 | 101.8 |  |  |
|  | 1000 | 5.6 | 103.1 |  |  |
| **Heart** | 1 | 5.6 | 105.7 | 1-1000 | 4 |
|  | 10 | 6.0 | 111.2 |  |  |
|  | 100 | 3.8 | 112.8 |  |  |
|  | 1000 | 5.1 | 103.8 |  |  |
| **Small Intestine** | 10 | 13.2 | 106.0 | 2-1000 | 20 |
|  | 50 | 3.4 | 87.8 |  |  |
|  | 100 | 14.6 | 107.2 |  |  |
|  | 1000 | 14.6 | 98.6 |  |  |
| **Brain** | 1 | 8.9 | 98.2 | 1-1000 | 4 |
|  | 10 | 4.4 | 105.5 |  |  |
|  | 100 | 4.7 | 104.8 |  |  |
|  | 1000 | 4.3 | 91.6 |  |  |

**Supplementary Table 3**. Parameters used in PBPK model for cross-species prediction

|  | **Value / Reference table** | **Source** |
| --- | --- | --- |
| **Physiochemical** | Table 2 | Experimental or Predicted by ADMET predictor |
| **Absorption of PDN**  P_eff_ (10^-4^ cm/s) | Rodent:1.27; Human:2.67 | Predicted by Gastroplus built-in regression model according to experimental PAMPA value (Table 2) |
| **Distribution** | |  |
| Plasma binding | Table 2 | Experimental |
| Tissue fraction unbound and Kp_uu_ (all tissues) | Supplementary Table 4 | Predicted by ADMET predictor or  fitted to mouse plasma and tissue concentration data |
| Permeability  (permeability-limited tissues) | Supplementary Table 5 |  |
| **Metabolic kinetic** | Supplementary Table 6 | Predicted by IVIVE according to experimental in vitro microsome and plasma clearance (Table 3) |
| **Renal excretion** | GFR*fup | Defaulted GFR for each species provided by Gastroplus database was used. |

**Supplementary Table 4. Unbound intracellular fraction in various tissues used in the PBPK model**

| **Tissue** | **Rodent** | | **Human** | |
| --- | --- | --- | --- | --- |
|  | **Nc** | **PD** | **Nc** | **PD** |
| Muscle | 0.08% | 2.90% | 0.05% | 3.70% |
| Spleen | 0.04% | 1.49% | 0.03% | 2.10% |
| Heart | 0.06% | 2.00% | 0.04% | 2.70% |
| Brain | 0.27% | 4.41% | 0.18% | 4.30% |
| Kidney | 0.03% | 0.95% | 0.02% | 1.40% |
| Rest | 0.04% | 1.49% | 0.03% | 2.10% |
| Adipose | 0.07% | 0.45% | 0.07% | 0.50% |
| Lung | 0.03% | 0.29% | 0.02% | 0.30% |
| Liver | 0.001% | 5.00% | 0.001% | 5.00% |
| Skin | 0.61% | 1.98% | 0.03% | 2.60% |
| Intestine | 0.003% | 1.50% | 0.003% | 1.50% |

**Supplementary Table 5. Unbound intracellular fraction and permeability surface product for intestine, liver and skin used in the PBPK model**

|  |  | **Cross-species** | **Mouse** | | **Hamster** | | **Human** | |
| --- | --- | --- | --- | --- | --- | --- | --- | --- |
| **Compound** | **Tissue** | **K_p,ex,uu_** | **K_p,ex_** | **F_u,ex_** | **K_p,ex_** | **F_u,ex_** | **K_p,ex_** | **F_u,ex_** |
| **PDN** | **Liver** | 4.57 | 1.00 | 0.57 | 1.00 | 0.57 | 1.30 | 0.53 |
| **Nc** | **Liver** | 304.86 | 0.97 | 0.44 | 0.97 | 0.44 | 1.11 | 0.44 |
| **PDN** | **Skin** | 7.92 | 3.50 | 0.28 | 3.40 | 0.29 | 4.28 | 0.28 |
| **Nc** | **Skin** | 1.357 | 0.380 | 0.005 | 0.380 | 0.005 | 0.362 | 0.006 |
| **Compound** | **Tissue** | **PStc/Vol** | **PStc (mL/s)** | **Vol (mL)** | **PStc (mL/s)** | **Vol (mL)** | **PStc (mL/s)** | **Vol (mL)** |
| **PDN** | **Liver** | 0.02 | 0.03 | 1.66 | 0.10 | 4.80 | 25.3 | 1254 |
| **Nc** | **Liver** | 0.00 | 0.00 | 1.66 | 0.00 | 4.80 | 0.227 | 1254 |
| **PDN** | **Skin** | 0.00 | 0.01 | 3.50 | 0.05 | 17.10 | 7.25 | 2537 |
| **Nc** | **Skin** | 0.01 | 0.03 | 3.52 | 0.17 | 17.10 | 24.8 | 2537 |
| **Nc** | **Intestine*** | 0.15 | 0.15 | 0.98 | 1.87 | 12.23 | 276 | 1800 |

*PStc of PDN in intestine is automatically generated by GastroPlus according to the PAMPA permeability based on their database.

**Supplementary Table 6. Scaled metabolic kinetic parameters for biotransformation of PDN and Nc in gut, plasma, and liver in human, hamster, and mouse microsomes or plasma**

|  | **Conversion** | **Scaling Factor** | **Mouse** | | **Hamster** | | **Human** | |
| --- | --- | --- | --- | --- | --- | --- | --- | --- |
|  |  |  | **In vitro** | **In vivo** | **In vitro** | **In vivo** | **In vitro** | **In vivo** |
| **Liver**  **V_max_ (mg/s/mg microsomes)** | PD - Nc | 1.54 | 0.00095 | 0.0015 | 0.0085 | 0.0130 | 0.0002 | 0.0004 |
|  | PD - Others | 1.56 | 0.14840 | 0.2310 | 0.1059 | 0.1649 | 0.0106 | 0.0164 |
|  | Nc - Others | 1.27 | 0.07474 | 0.0949 | 0.1152 | 0.1463 | 0.0581 | 0.0738 |
| **Gut***  **V_max_ (mg/s/mg microsomes)** | PD - Nc | 0.05 | 0.00095 | 0.00004 | 0.0085 | 0.00040 | 0.00024 | 0.00001 |
|  | Nc - Others | 0.55 | 0.07474 | 0.0408 | 0.1152 | 0.0629 | 0.0581 | 0.0317 |
| **Plasma**  **k (1/s)** | PD - Nc | 1.15 | 0.0058 | 0.0067 | 0.0045 | 0.0052 | 0.00026 | 0.00023 |
|  | PD - Others | 1.30 | 0.0062 | 0.0080 | 0.0146 | 0.0189 | 0.0022 | 0.0068 |

*In vivo gut metabolic kinetic parameters were predicted based on the in vitro liver parameters.

**Supplementary Table 7. Kinetic solubility assay controls results**

| **Controls** | **Average Solubility (µg/mL)** |
| --- | --- |
| Albendazole | <1 |
| Phenazolpyridine | 26 |
| Furosemide | >66 |

**Supplementary Table 8. PAMPA permeability controls results**

| **Controls** | **Average Permeability**  **(10^-6^ cm/s)** |
| --- | --- |
| Ranitidine | <1 |
| Dexamethasone | 122 |
| Verapamil | >1000 |
